# Cholera transmission dynamic models for public health practitioners

**DOI:** 10.1186/1742-7622-11-1

**Published:** 2014-02-12

**Authors:** Isaac Chun-Hai Fung

**Affiliations:** 1 Department of Epidemiology, Jiann-Ping Hsu College of Public Health, Georgia Southern University, Statesboro, GA, USA; 2 Health Economics and Modeling Unit, Scientific and Program Services Branch, Division of Preparedness and Emerging Infections, National Center for Emerging and Zoonotic Infectious Diseases, Centers for Disease Control and Prevention, Atlanta, GA, USA

## Abstract

Great progress has been made in mathematical models of cholera transmission dynamics in recent years. However, little impact, if any, has been made by models upon public health decision-making and day-to-day routine of epidemiologists. This paper provides a brief introduction to the basics of ordinary differential equation models of cholera transmission dynamics. We discuss a basic model adapted from Codeço (2001), and how it can be modified to incorporate different hypotheses, including the importance of asymptomatic or inapparent infections, and hyperinfectious *V. cholerae* and human-to-human transmission. We highlight three important challenges of cholera models: (1) model misspecification and parameter uncertainty, (2) modeling the impact of water, sanitation and hygiene interventions and (3) model structure. We use published models, especially those related to the 2010 Haitian outbreak as examples. We emphasize that the choice of models should be dictated by the research questions in mind. More collaboration is needed between policy-makers, epidemiologists and modelers in public health.

## Introduction

Since the 19th century, humans have experienced seven cholera pandemics. The seventh pandemic started in Indonesia in 1961 and continues to threaten vulnerable populations globally [1]. The cholera outbreak that began in October 2010 in Haiti, where cholera had been absent for a century, reminds us the importance of timely cholera prevention, treatment and control and the critical importance of water and sanitation infrastructure that has eliminated cholera from much of the developed world [2].

To better understand cholera epidemiology retrospectively and to predict the impact of interventions in the future, many researchers have begun using mathematical models as tools complementary to field epidemiology and statistical analysis. Mathematical models help us conceptualize the transmission dynamics in a quantitative way and allow us to test different hypotheses and understand their relative importance *in silico*. Important epidemiological observations and hypotheses for cholera have been modeled; examples include estimation of the basic reproduction number (R_0_) [3,4], seasonal variation in cholera incidence [5], inapparent cholera infections [6], hyperinfectivity of *V. cholerae*[7], human-to-human transmission [8], and the role of human mobility and river networks in transmission [5,9]. Mathematical models also allow us to prospectively estimate the impact of various interventions, from treatment (oral rehydration therapy and antibiotics) to prevention (oral cholera vaccine (OCV), and water, sanitation and hygiene (WASH) interventions (e.g. [9-12])).

The purpose of this paper is to introduce cholera dynamic transmission models to public health practitioners, with an educational emphasis of conveying modeling concepts to students of these models. Models are simple, but not simplistic representations of the real world. They are used to capture the “essence” of a complex phenomenon. Models may help us better understand the relationship between different parts of the system. Some models may shed light on past epidemics while some may help us forecast the future. Here we define dynamic transmission models as models that explicitly simulate the transmission dynamics of infectious diseases in time. This paper will focus on the ordinary differential equation (ODE) models (population-based continuous-time models as contrast to population-based discrete-time models using difference equations), while we will mention relevant agent-based models where appropriate (e.g. [9]).

Through a basic model, we will explain the major parameters and how interventions may change them. We will discuss how different assumptions and hypotheses can be accommodated by making changes to the model’s structure. Focus is given to the way different research questions dictate the model structure. Published models were chosen as illustrations and the list is not meant to be exhaustive. Priority is given to papers that model specifically the 2010 Haiti cholera epidemic. Instead of being a systematic review of all existing cholera models, my aim is to highlight three current major challenges of modeling efforts of cholera transmission dynamics: (1) parameter uncertainty and model misspecification; (2) interventions (especially, water, sanitation and hygiene); and (3) model structure. Spatial and climatic elements are also important features but they are beyond the scope of this paper (they are briefly discussed in the Additional file 1). For a detailed review of the recent cholera modeling literature, please refer to ref. [13].

## Some basic concepts

First, let us review some basic concepts. In an ODE model of infectious diseases, we divide the population into a number of compartments. For example, in a Susceptible-Infected-Recovered (S-I-R) model, the population is divided into three compartments depending on their status of being susceptible to the infection (S), being infected and infectious (I), and having recovered from the infection (R). Individuals in each compartment were assumed to be homogeneously mixing with each other [14]. The ODEs of the model capture the change of the number of individuals in each compartment over continuous time. While ODE models have their own set of assumptions and limitations, they are commonly used in epidemiologic modeling because we can use a few equations to represent the transmission dynamics and create an easy-to-understand model for public health practice.

The basic reproduction number, R_0_, is usually defined as the number of individuals that an infected (and infectious) individual can infect when he or she is introduced into a completely susceptible population. For example, for a disease with R_0_ = 2, an infected individual on average infects two individuals in a totally susceptible population. The effective reproduction number, R or R_E_, is defined as the number of individuals infected by a typical infectious individual when a fraction of the population is protected from infection through immunity, prophylaxis or non-pharmaceutical interventions [15]. For example, for a disease with R_0_ = 2, and if half of the population is immune to this disease, R_E_ = R_0_ * ½ = 1.

ODE models can be programmed in computers using different languages, software and platforms, for example, C, C++, Matlab, Mathematica, R, and Berkeley Madonna. For further details of these models, public health students of mathematical modeling may refer to general modeling texts, for example, Anderson and May [16], Cummings and Lessler [14], Keeling and Rohani [17], and Vynnycky and White [15].

## The basic model

Following the example of Grad et al. [18], we adapt the model of Codeço [19] as our basic model through which we explain how the transmission dynamics of cholera is modeled mathematically.

**Figure 1:**
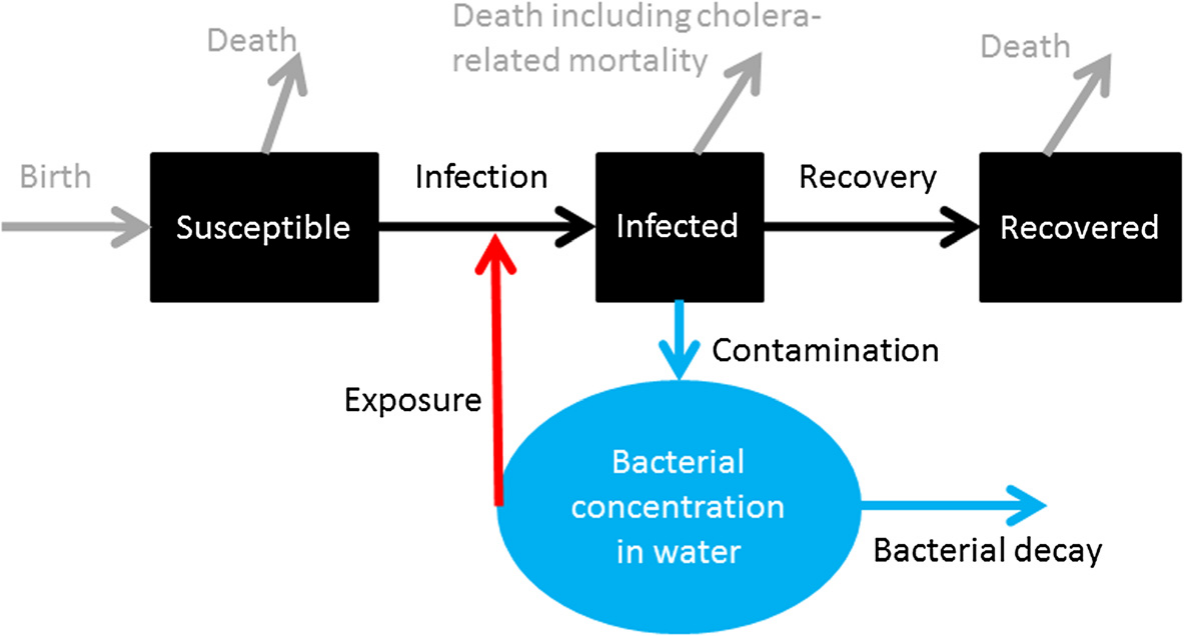
**Schematic of a basic model of cholera transmission dynamics (model adapted from Codeço **[19]**).**

Figure 1 presents a schematic of the basic model. The black boxes represent people: susceptible (S in equations in “The basic model” in Additional file 1); infectious (I); and recovered (R). The blue circle represents cholera bacterial concentration in the water reservoir (B).

• Black arrows: Susceptible people become infected/infectious and they later recover and become immune.

• Blue arrows: Infectious people contaminate the water supply with bacteria and the bacteria decay.

• Red arrow: Susceptible people are exposed to contaminated water and may become infected.

• Gray arrows: People are born into the susceptible population; they may die as a result of cholera infection or other reasons.

Please refer to the Additional file 1 for the equations and explanations of the variable and parameters. For models that simulate an outbreak within a short period of time (e.g. one year), one can ignore the dynamics of population growth (birth rate and death rate, gray arrows) and assume a constant population.

Here are the key assumptions:

1. Infected individuals are infectious and contribute to bacteria shedding, which imply that asymptomatic individuals contribute as much bacteria to the water supply as symptomatic individuals.

2. Immunity obtained through infection lasts longer than the timeframe studied by the model (for example, 1 year).

These assumptions will be relaxed later as we modify the model structure to accommodate asymptomatic individuals and waning immunity.

In the following sections, we will discuss three current major challenges of modeling efforts of cholera transmission dynamics: (1) parameter uncertainty and model misspecification; (2) interventions (especially, water, sanitation and hygiene), and (3) model structure.

## Model misspecification and parameter uncertainty

The first challenge is model misspecification and parameter uncertainty, that was highlighted by Grad et al. [18] and is briefly summarized as follows. To parameterize a cholera transmission model is challenging. In the basic model, we note that the rate of cholera transmission is a product of the force of infection (λ) and the size of the susceptible population (S). The force of infection in turn depends on three parameters or variables (see Additional file 1):

1. β: the “contact rate” between the susceptible population with contaminated water,

2. B: the level of contamination of the water supply (*V. cholerae* concentration), and

3. κ: the concentration of *V. cholerae* at which the infection rate is 50% of the maximum infection rate, that is β.

### Model mis-specification

The “contact rate” and the *V. cholerae* concentration are largely unknown in most contexts. As Grad et al. [18] have rightly pointed out, there are no simple methods that can convert results of experimental studies (for example, “a measured dose-response relationship between number of vibrios ingested and the risk of infection” [18]) into the “contact rate” between susceptible individuals and bacteria in water (β), and the concentration of *V. cholerae* in the water reservoir that will make 50% of the susceptible population ill (κ) [18]. The rate at which susceptible individuals become infected is determined by many variables in reality, most of which cannot be easily measured. As the “contact rate” (β) can rarely be measured directly from experimental studies, it is usually estimated by fitting models to time series data. These problems are referred as *model mis-specification*, where the item of interest is different from what the model actually models, e.g. empirical experiments provide dose data in terms of the number of bacteria, while the model needs the bacteria concentration data in the environmental water [18].

### Parameter uncertainty

The per capita recovery rate is probably the most certain of all parameters in the model. It is approximately equal to the reciprocal of the duration of infection (1/γ), a parameter that more data are available. Cholera life span in water reservoir (1/δ) depends on the local environment. While it is largely unmeasured in many endemic or epidemic contexts, modelers can use historical experimental data from the literature and therefore this parameter is also relatively certain. The rate of water contamination by infectious people shedding *V. cholerae* into the water reservoir (ξ) depends on both bacteria shedding of the infected individuals (a biological quantity) and the level of sanitation in the environment (an environmental assessment). This is largely unknown in most contexts. These problems are that of *parameter uncertainty.*

Therefore, in the cholera mathematical model literature, the values of the parameters used vary greatly as seen in Table 1. Grad et al. have cautioned potential users of cholera models in their interpretation of modeling outputs as the high variability of some of these parameters would translate into great uncertainty in the outputs [18]. Uncertainty analysis should be performed for these parameters [18]. (For a detailed analysis of parameter uncertainty of cholera models, please refer to ref. [18]. For a discussion of the values and their data sources of some of the parameters, see Additional file 1).

**Table 1:** **Parameters assumed or fitted based on selected published mathematical models of cholera (partly adapted from Grad et al., 2012 **[18]**)**

**Symbol**	**Parameters**	**Range**	**Comments**	**Potential data from field epidemiology**
β	Rate of “contact” with reservoir water (days^-1^)	10^-5^ to 1	Difficult to convert empirical data into this “contact” rate.	Identity and location of drinking water sources; frequency of water usage and volume drawn from these sources
1/γ	Duration of cholera infection (days)	2.9 to 14	The most certain among the 5 parameters	Clinical data
1/δ	Cholera life span in water reservoir (days)	3 to 41	Usually not measured; depending on local environment (temperature, salinity), nature of the water source (running or static), cholera phage concentration. Historical experimental data available.	Water samples for microbiological experiments
ξ	Rate of water contamination by humans, i.e. rate of increase in *V. cholerae* concentration in the water reservoir (cells * mL^-1^ * person^-1^ * day^-1^)	0.01 to 10	Usually not measured; depending on infection severity, sanitation provision and water reservoir size.	Clinical data: frequency and volume of watery stool and especially concentration of vibrios in watery stool.
κ	Concentration of cholera that yields 50% chance of infection (cells/mL)	10^5^ to 10^6^	The dose–response curves depend on strain and biological context (e.g. gastric acidity). While empirical data provided data for doses (number of bacteria), the parameter measures in concentration.	Based on the volume of water intake per person per day and the vibrio concentration in the water samples, one can estimate the dose of vibrio intake per person per day

Equally important is data collection from the field that informs model parameterization (see Table 1). For example, in a neighborhood affected by cholera, we can investigate the various sources of drinking water for a given household, their relative importance in terms of volume drawn or frequency used, and the concentration of cholera vibrios and their decay rate in water samples collected from these sources. Just as the human contact data for constructing the contact matrix between different age groups in a population is important for influenza transmission models [20], collecting water usage data from a community is important to the parameterization of people’s “contact” rate with contaminated water. Eisenberg, Robertson and Tien [21] recently suggested that if we can measure pathogen persistence time in environmental water sources (δ) or pathogen concentration in the water (B), we can better estimate the parameters of the waterborne transmission pathway.

## Interventions

The second challenge is to model interventions correctly. Interventions can be represented in the model as a change in the value of a parameter, or a change in the model structure. I will first discuss treatment, and then OCV, followed by WASH interventions.

## Treatment

The primary treatment for a cholera patient is oral rehydration treatment (ORT). It prevents dehydration and averts mortality [22]. Severe cases are given antibiotics to speed up their recovery and to reduce the amount of bacteria shed into the environment (see ref. [23], p.127). The effect of antibiotics treatment can be simulated in a model by increasing the recovery rate, γ, and by reducing the rate of water contamination by treated patients in terms of *V. cholerae* concentration in the water reservoir, ξ [11]. Another model simulated combined ORT with antibiotic treatment by decreasing cholera-related death rate and increasing recovery rate [24]. Alternatively, patients under treatment can be represented by a distinct compartment [25]. In this case, there will be a rate at which infected patients receives treatment and the recovery rate of the treated patients respectively.

## Vaccine and immunity

**Figure 2:**
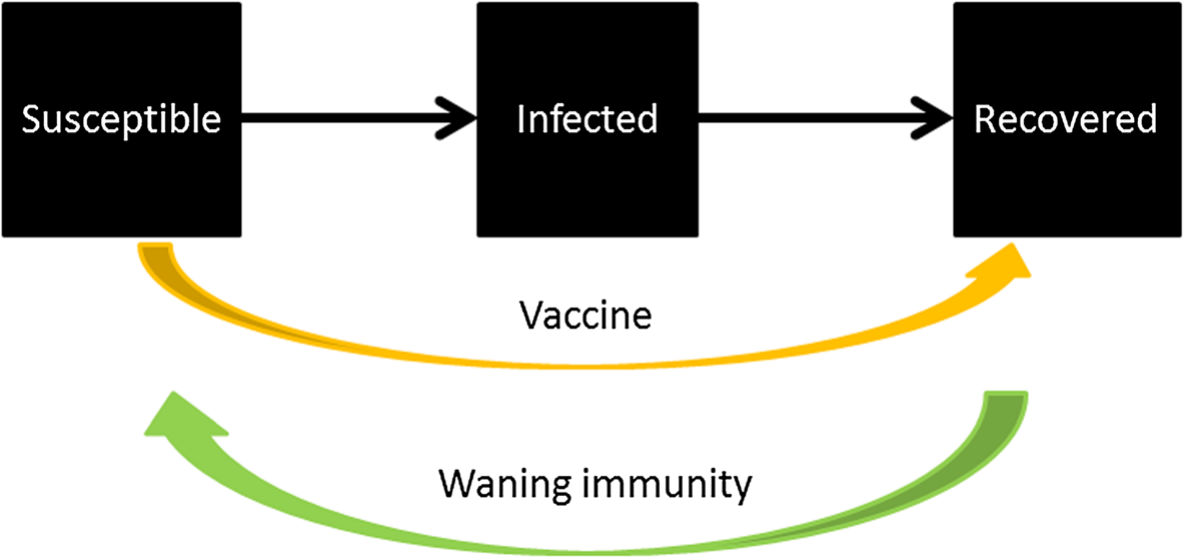
Vaccine and waning immunity (Model 1).

People recovered from cholera develop immunity that protects them from being infected again for several years [1]. OCV, if completed with the adequate doses (2 doses for either Dukarol or Shanchol), can also immunize individuals against cholera infection for several years before they become susceptible again [26-29]. A simple way to represent it in the basic model is to allow oral cholera vaccine transferring people from the Susceptible compartment (S) to the Recovered/Immune compartment (R) (Figure 2, orange arrow, as in [24]). Similarly, as immunity wanes, people are transferred from the Recovered compartment to the Susceptible compartment (Figure 2, green arrow). If infection-conferred immunity wanes at a rate different from that of vaccine-conferred immunity, then a separate compartment representing vaccinated individual is preferred.

Not everyone vaccinated will be immune to infection. (For example, Shanchol confers 65% direct protection against cholera in a 5-year follow-up period) [29]. Furthermore, there is an indirect effect through which unvaccinated individuals are protected in communities where some individuals are immunized. The concept of herd immunity refers to the fact that individuals immune to an infection will not transmit an infection since they are not infected in the first place. Therefore, by vaccinating individuals in a population, indirect protection is conferred to other members of the population who are not immunized. For non-immunizing interventions like water, sanitation and hygiene interventions, a similar concept may apply and is sometimes known as herd protection or indirect protection.

**Figure 3:**
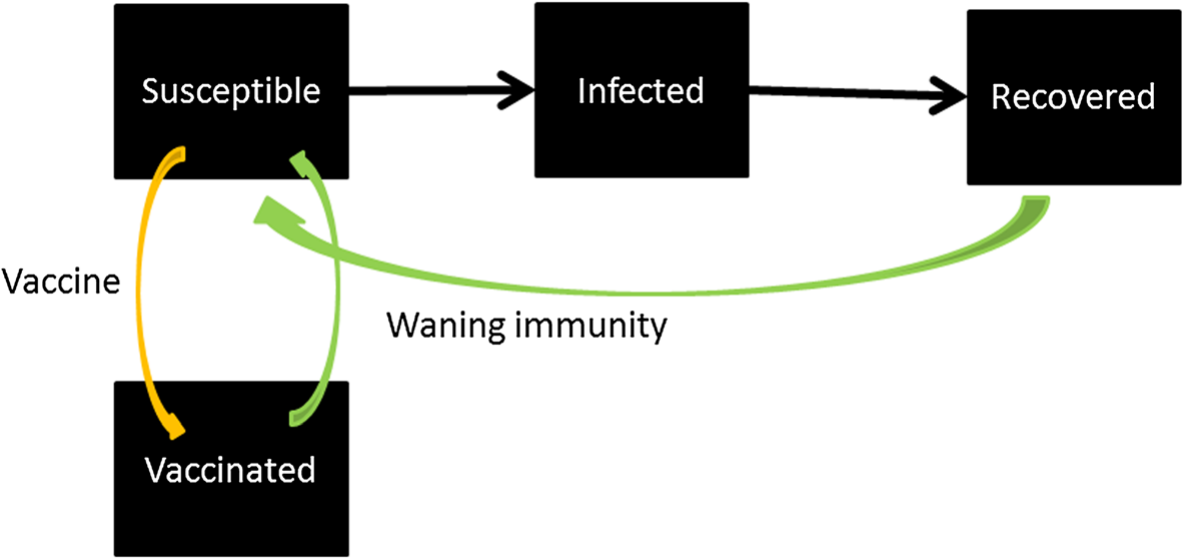
Vaccine and waning immunity (Model 2).

A dynamic model that explicitly simulates the transmission mechanism can take these factors into account, if we slightly modify the model structure as in Figure 3. The compartment “Vaccinated” represents individuals who receive two doses of vaccine, are successfully immunized and are truly immune. Some susceptible individuals become immune through vaccination (orange arrow) or through recovery from infection (black arrow). As their immunity wanes, they become susceptible again (green arrow). For more discussion, see Additional file 1.

## Water, sanitation and hygiene interventions

**Figure 4:**
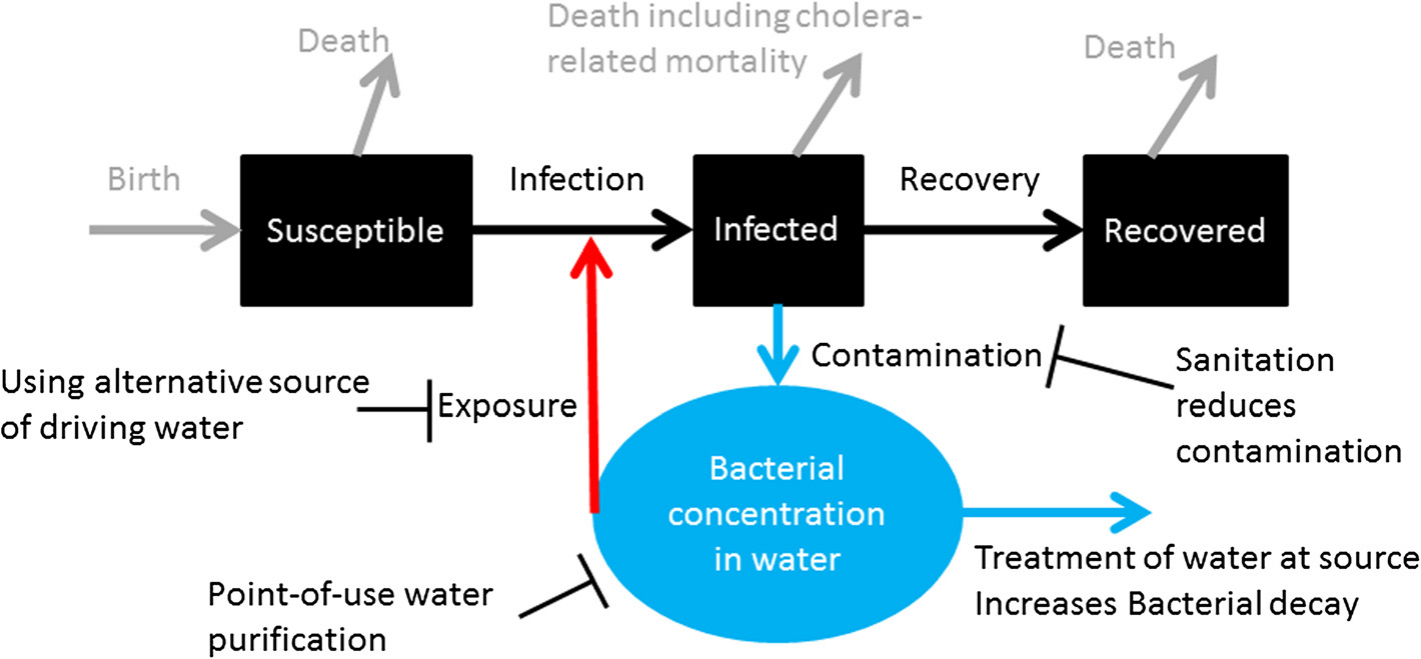
Water, sanitation and hygiene interventions.

Provision of clean water, sanitation and personal hygiene are all important interventions that can stop cholera transmission. In transmission dynamic models, one can simulate the effects of these interventions by changing the values of one or more parameters. Sanitation interventions, from latrines to flush toilets, reduce water contamination from human feces by separating them from the drinking water supply (reducing contamination rate, ξ). Chlorination of piped water removes bacteria from the water (increasing the removal rate of bacteria, δ). Point-of-use purification via boiling, chlorination, or filters, reduces the bacterial concentration in drinking water (reducing B). Interventions that promote alternative sources of drinking water reduce “contact” between susceptible populations and contaminated water (reducing β, as in [10-12,24]) (Figure 4, Table 2 and Additional file 1). Sometimes intervention descriptions in the modeling literature may be confusing, for example, provision of clean drinking water might be described as “sanitation” as in ref. [12,24] (see Additional file 1: Table S3). Apparently, linking coverage to effectiveness is not easy: some models might reduce β by a prescribed fraction in the absence of any inputs in coverage and in protective effectiveness of a given intervention (Table 3). Instead of simply reducing β by x%, it may be more useful to model the provision of clean water with protective effectiveness, y%, and coverage, z%, that results in x% reduction of β, i.e. x = y * z, assuming a linear coverage-effectiveness relationship. Policy-makers need to know which types of WASH interventions to choose and the level of coverage needed to obtain desired outcomes (as in number of averted cases of cholera).

**Table 2:** Effect(s) on model parameters by water, sanitation and hygiene (wash) interventions

**WASH interventions**	**Effect(s) on parameters**
Sanitation interventions and health promotion of their utilization	Reduce water contamination rate (ξ)
Treatment of water at source (e.g. chlorination of piped water)	Increases the rate of bacteria removal from water (δ)
Point-of-use water purification (via boiling, chlorination or filters)	Reduces the concentration of bacteria (B) of drinking water
Using alternative source of drinking water	Reduces the “contact” rate between susceptible population with contaminated water (β)

**Table 3:** Reduction in transmission coefficient (“contact rate”) by water, sanitation and hygiene (WASH) interventions in selected published models of the Haiti epidemic

**Model**	**WASH intervention that the model was supposed to simulate**	**Reduction in transmission coefficient (“contact” rate, β)**	**Empirical data sources for WASH interventions’ effectiveness or coverage**
Andrews and Basu [11]	Expansion of clean water provision	Exponential decline in β (1% decrease per week)	Estimated coverage of clean water since the outbreak’s beginning, from two progress reports by Red Cross and Oxfam respectively
Bertuzzo et al. [12]	Sanitation: “a set of measures”, not explained in their paper	40% reduction for 1 month	None provided
Chao et al. [9]	Educational campaign to promote improved hygiene and sanitation, that accompanies the vaccination campaign	10% or 30% (additional) reduction, in areas covered by vaccination campaign	None provided
Tuite et al. [10]	Clean water provision, either to “the same number of people who could be vaccinated” or to “the number of people who would need to receive clean water to have the same effect on epidemic spread as that achievable through vaccination”	Reduction of waterborne transmission (but not human-to-human transmission) by a fraction that is the probability of provision of clean water within a Haitian *department* (equivalent to a province), for up to 2 years, beginning at the same time as vaccination program would do for the sake of comparison.	None provided. Implied assumption: 100% reduction of “contact” rate if covered by clean water provision.

Probably the weakest link in modeling WASH interventions is the dearth of data that link the programmatic variables (e.g. implementation coverage) to the reduction of the transmission coefficient. For example: in one paper [24], while a value of 10 US dollars per the square of level of sanitation was provided, it would be in the interest of the readers to provide the means to convert such “level of sanitation” (i.e. the proportion of reduction in β) into any quantity of coverage of any sanitation projects in reality. Likewise, it will be beneficial to the readers if details can be provided as to the “set of measures” of sanitation that would lead to a 40% reduction in β over a period of one month in Haiti in another example [12]. Similarly, readers would benefit if a third example [9] could provide data to support their choice of 10% or 30% reduction in cholera exposure through a health education campaign of hygiene and sanitation that accompanies the vaccination campaign.

There are exceptions though. One model [11] simulated “the effect of a 1% per week reduction in the proportion of the population consuming contaminated water based on present estimates of clean water provision” in Haiti, by converting “the estimated proportion covered [by clean water provision] since the start of the cholera outbreak into a rate of [increasing] clean water provision”. Two progress reports published by Red Cross and Oxfam respectively were cited as references. Such a rate of increasing clean water provision, as a daily percent reduction of the rate of drinking contaminated water (β), led to an exponential decline of β [11]. This implies that as coverage of clean water provision increases in time (number of weeks, n), the “contact” rate with contaminated water (β) would reduce as: β*(1–0.01)^n^. But it is difficult to tell how much more coverage increase per day is needed to achieve such an effect.

Another model [10] estimated the number of people who would need clean water provision to achieve the same effect as 500,000 people being vaccinated in Haiti. The implied assumption was that if clean water was provided, there would be a 100% reduction of waterborne transmission (but not human-to-human transmission). What mattered was coverage. (See section ‘Hyperinfectious bacteria and “human-to-human” transmission’ below.)

The WASH interventions that are chosen, and their effectiveness and coverage have a huge impact upon the results. Comparing a poorly defined WASH intervention with OCV could inadvertently misinform policy-makers about which programs should be expanded.

While it is useful to illustrate ranges of possibilities, future studies should be designed to provide data to parameterize these models. Another example was a model that incorporated a separate compartment for people who received health education and therefore may be infected at a rate different from those who did not. It will be beneficial if empirical data can be provided to parameterize the rates of health education, of failure to comply with instructions of health education, and of infection rates of health-educated individuals (all three parameters were “assumed”) [25]. Likewise, for the compartment for quarantine of health-educated individuals who were exposed to cholera, their rate of quarantine after exposure and their rate of actually being infected, it will be beneficial if empirical data can be provided to parameterize them [25].

## Model structure: additional components

The third challenge is to correctly build the model structure. There are debates in the literature as to the essential components of a model that successfully replicate observed cholera dynamics. These are tied to our understanding in biology and epidemiology as to the relative importance of certain features of the cholera life cycle or its epidemiology. The basic model can be modified to take these elements into account. In this section, we focus on two issues: (1) asymptomatic, or ‘inapparent’, infections, and (2) hyperinfectious bacteria and human-to-human transmission.

## Asymptomatic infection

There was a debate with regard to the relative importance of asymptomatic infection to transmission dynamics [30]. As noted by Grad et al. [18], the basic model assumes that throughout an epidemic, there is a constant ratio of asymptomatic to symptomatic infections, and that the infectious dose “determines the likelihood of infection, but not the likelihood of being symptomatic” [18]. However, the volume of bacteria shedding is likely very different: A person with severe cholera shed a lot more stool than that shed by an asymptomatically infected person. Rate of diarrhea for severe cholera cases is as high as 500–1000 mL/h [31]. Severe cases may shed bacteria for one to two weeks while asymptomatic patients typically shed for one day [32]. It is also likely that the amount of viable *Vibrio cholerae* per gram of stool excreted by a symptomatically infected cholera patient is greater than (or equal to) the amount of viable vibrios per gram of stool excreted by an asymptomatically infected person. It is perhaps worth noting that for the most part, surveillance data only captures symptomatic infections.

**Figure 5:**
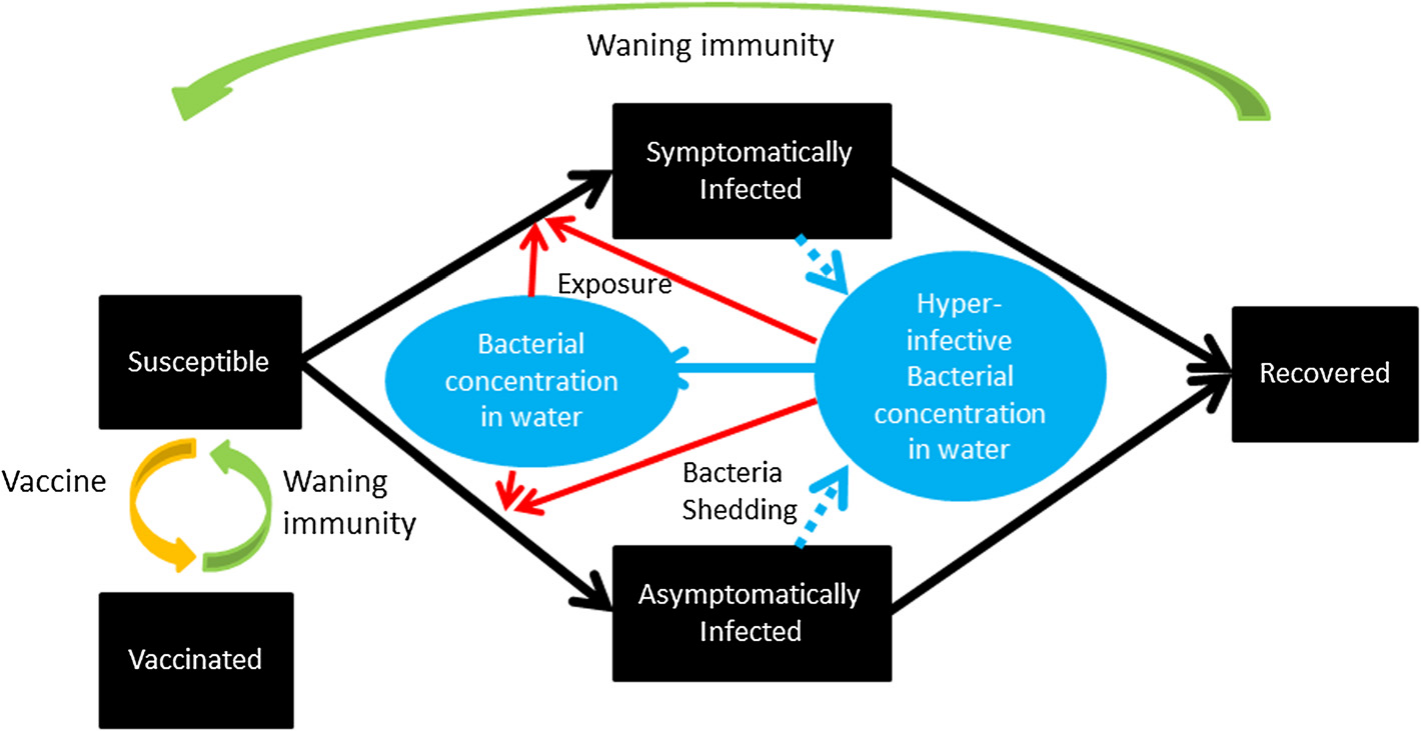
**Hyperinfectious bacteria and asymptomatic infection (adapted from Andrews and Basu, 2011 **[11]**) note: The “Vaccinated” compartment refers to successfully vaccinated individuals who become immune.**

Bertuzzo et al. [12] took into account asymptomatic infection when they compared their simulation results to the observed cumulative incidence curve, including an underreporting scaling factor (See Additional file 1), but chose not to distinguish asymptomatic infections from symptomatic infections in the mathematical model structure. Other modelers believe that these are important elements and incorporated into their model a compartment for asymptomatic individuals whose bacterial shedding rate are lower than that of symptomatic individuals (60% - 90% of infected individuals being asymptomatic as in ref. [11], see Figure 5), less infectious than symptomatic individuals (e.g. 10% of the infectiousness of symptomatic individuals as assumed in ref. [9]), and with a lower cholera-related death rate and faster recovery rate (as in ref. [24]). Chao et al. [9] found that their results were sensitive to the fraction of infected people who became symptomatic. The higher the symptomatic proportion, the higher was the incidence of reported cases. Others proposed that ‘inapparent’ infections may prove to be like a vaccine, through which people acquire immunity against cholera [6]. This idea is consistent with experimental data from a volunteer challenge study [33].

The key to the debate in ref. [30] was how would we explain the rapid reduction of the effective reproduction number in the first few months of the Haitian outbreak. Underreporting of cases, including asymptomatic cases, should be taken into account when fitting modeling outputs to observed data (even if the model does not have a distinct compartment for asymptomatic cases). Nonetheless, the reduction in effective reproduction number during the first three months of the epidemic cannot be solely explained by the depletion of susceptible individuals through infection, as the surge in incidence in June and July 2011 (see Figure 1 of ref. [34]) would be difficult to explain. (For details of the debate and our comments, see Additional file 1).

## Hyperinfectious bacteria and “human-to-human” transmission

The second issue is how important hyperinfectious *V. cholerae* are to the transmission process. A decade ago, Merrell et al. [35] discovered that freshly shed *V. cholerae* were much more infectious than those that were grown in-vitro. However, these hyperinfectious bacteria would lose their hyperinfectiousness once they were cultured in vitro in broth for 18 hours. Later, researchers demonstrated that mouse-passaged *V. cholerae* also demonstrated similar hyperinfectious properties as those freshly shed by humans, but such properties would disappear after 24 hours in the *in vitro* environment [36]. It has also been demonstrated that growth in a biofilm induces a hyperinfectious phenotype of *V. cholerae*[37]. This was the basis of the hypothesis that freshly shed *V. cholerae* existed in a hyperinfectious state for less than one day and that they contributed to cholera transmission more than we previously expected. These implied that a so-called “human-to-human” transmission route played an important role than the environmental, “water-borne” route [38].

**Figure 6:**
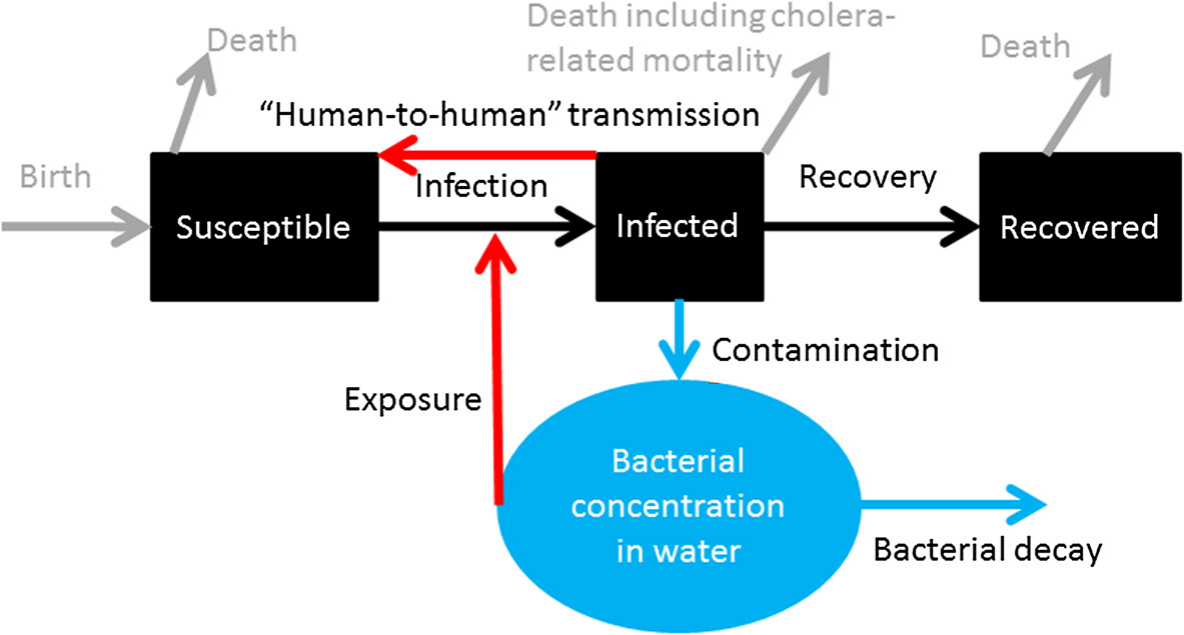
“Human-to-human” infection incorporated into the basic model.

Some modelers argue that these hyperinfectious bacteria hold the key to our understanding of cholera transmission dynamics (e.g. refs. [7,11]) (Figure 5). They include in their models a separate compartment for these hyperinfectious bacteria with a very high infectiousness (a higher β). These bacteria will leave their hyperinfectious state and become normal within a day (Table 4). However, Pascual et al. [39] rightly argued that the extra compartment is redundant for most purposes unless the specific question in mind is to study the hyperinfective state. Therefore, for the sake of parsimony, “human-to-human” transmission is seen by some as a good proxy for the impact of hyperinfectious bacteria, as in refs. [10,40,41] (Figure 6; see Additional file 1 for details). With their very brief period of existence, the impact of hyperinfectious bacteria is the greatest upon family members who live under the same roof and share the same water source. This is consistent with observations in a cholera outbreak that family contacts of an index case had a higher risk of getting infected [42]. Therefore, Chao et al. limited “human-to-human” transmission to transmission within a household in their model [9].

**Table 4:** **Parameters for hyperinfectious bacteria as found in selected published mathematical models (adapted from Grad et al., 2012 **[18]**)**

**Parameter**	**Andrews and Basu **[11]	**Hartley et al. **[7]	**Chao et al. **[9]	**Original empirical data sources cited by authors**
Multiplier for infectiousness of freshly shed vibrio (hyperinfectious state)	50	700	100	[35,36]
Duration of hyperinfective state (hours)	24	5	24	[35,36]

For further discussion on these parameter values, please refer to the Additional file 1.

To model “human-to-human” transmission is challenging in two aspects. Firstly, the relative magnitude of the transmission coefficient (“contact” rate) of “human-to-human” transmission to waterborne transmission is uncertain (See Table 4 and the Additional file 1). Secondly, to correctly capture the impact of interventions upon “human-to-human” transmission is not easy. Take for example, in Tuite et al.’s model [10], the relative reduction in total cases by “equal allocation of clean water” was much smaller than that by an “optimized allocation of vaccine”. The major reason was that Tuite et al. assumed that clean water provision stopped waterborne transmission but not “human-to-human” transmission. However, clean water provision may, in fact, reduce the “human-to-human” transmission. Cholera is transmitted via the oral-fecal route. The hyperinfectious state only makes the necessary infectious dose (or the IC_50_) much lower. Given that the “human-to-human” transmission is only a mathematical proxy of the impact of the hyperinfectious bacteria, clean water provision should have an impact on human-to-human transmission, even if it may not stop transmission completely.

## Moving forward

Our research questions dictate our choice of models. For the purpose of public health practice and policy-making, we propose the following two directions for future development of cholera models.

The first direction is emergency preparedness and response for cholera outbreaks. During the early phase of the Haitian epidemic in 2010, the US Centers for Disease Control and Prevention (CDC) made use of Abrams et al.’s model [43] to inform policy-makers (that model will be further discussed in the Additional file 1). In the future, we can cross-validate models for both their model structure and parameters against various historical epidemiological datasets, and then use the validated models for outbreak response. In some outbreak scenarios, seasonality can be omitted from the model, as only a short time frame is needed. Elements of spatial heterogeneity can be included if relevant data are readily available. Modeling packages that use models of relatively few parameters and variables can be created and made readily available before the next outbreak. At the beginning of an outbreak when data are limited, field epidemiologists and policy-makers (for example, Epidemic Intelligence Service officers and their superiors in the CDC) who are not trained in mathematical modeling can deploy such models to provide estimates of attack rates (cumulative incidence) and intervention effects in different scenarios. The model inputs will either be provided for by the model as default (obtained from historical data in the literature) or require users’ inputs (as estimated based on limited data at the beginning of an outbreak). To facilitate its use in developing countries, the use of software that requires expensive licenses can be avoided. Free software like R is a good alternative. Many public health practitioners find the availability of a user-friendly Graphical User Interface helpful. One example is to use Excel as the user’s interface to an executable file compiled from a C++ code, as in the influenza model Community Flu 2.0 that is available on CDC website [44].

The second direction is cholera control in endemic contexts. First, the elucidation of the drivers of, and their effects upon, seasonal patterns of cholera incidence, and the effect of population and hydraulic movements upon spatial heterogeneity of incidence, will help epidemiologists predict future outbreaks (some of the related models are briefly discussed in the Additional file 1). Second, the estimation of the long-term effects on cholera incidence and the return on investment of long-term infrastructure building and intervention programs will be valuable to policy-makers. Complementary to this modeling effort, we will need to collect better data for intervention effectiveness (including indirect effect) and costs.

## Conclusion

Dynamic transmission models of cholera have been developed very rapidly in recent years, especially after the 2010 Haitian outbreak. Many models have been published but few make any impact on decision-makers and field epidemiologists. This paper provides an introduction to the basics of ordinary differential equation models of cholera transmission dynamics, in the hope that the usefulness of modeling in public health research and decision-making may be better appreciated. Field epidemiologists are crucial in the partnership with modelers as they provide actual data that help parameterize the models. Model-driven data collection and data-driven model construction are equally important. Likewise, policy makers that are well-informed with the assumptions and implications of mathematical models and the data that are used to parameterize them, will be able to use mathematical modeling studies to facilitate their decision-making. More collaboration between policy makers, epidemiologists and modelers is needed if we want to make progress in controlling cholera in Haiti and beyond.

## Competing interests

The authors declare that they have no competing interests.

## Supplementary Material

Additional file 1 Online Supplementary Materials. Click here for file
