# Supplementary material for: Cholera transmission dynamic models for public health practitioners
Source: Emerg Themes Epidemiol. 2014 Feb 12;11:1. doi: 10.1186/1742-7622-11-1 (PMC3926264; doi:10.1186/1742-7622-11-1)
Supplement: Additional file 1 — Online Supplementary Materials. [file 1742-7622-11-1-S1.pdf]

# CHOLERA TRANSMISSION DYNAMIC MODELS FOR PUBLIC HEALTH PRACTITIONERS

**Additional File 1**

**Online Supplementary Materials**


**Isaac Chun-Hai Fung, PhD**

Email: CFung@georgiasouthern.edu


Disclaimer: The findings and conclusions expressed in this report do not necessarily represent the official position of the Centers for Disease Control and Prevention.

## CONTENTS


Introduction ........ 2
The basic model ........ 2
The force of infection ........ 3
Summary of selected models ........ 3
Model misspecification ........ 5
Parameter uncertainty ........ 5
Duration of cholera infection ........ 5
Cholera life span in water reservoir ........ 6
Comparison of values for key parameters across selected models ........ 7
Vaccination ........ 9
Vaccination Strategy ........ 10
Vaccine Effectiveness ........ 10
Water, sanitation and hygiene ........ 12
Asymptomatic infections ........ 12
Hyperinfectious bacteria and "human-to-human" transmission ........ 13
Choice of parameters ........ 13
Spatial elements: human movement & river network ........ 14
Climate & seasonality ........ 15
Cholera modeling applied in an outbreak scenario: Abrams et al. as an example ........ 16
References ........ 18

# INTRODUCTION

The online supplementary materials complement the main text with more detailed analyses and discussion on certain topics. The materials are arranged in a sequence similar to that in the main text for easy reference.

One of the earliest mathematical models of cholera dynamics was probably that of Capasso and Paveri-Fontana [1]. It was a model of 2 ordinary differential equations, fit to data of the cholera outbreak in Bari, Italy in 1973. Codeço explicitly acknowledged that her model [2] was the extension of ref. [1].

Another early model of cholera transmission was that of Cvjetanovic, Grab and Uemura, as described in Chapter 5 (pages 65-79) of their modeling "textbook", *Dynamics of acute bacterial diseases. Epidemiological models and their application in public health*, which was a supplement to the Bulletin of the World Health Organization in 1978 [3, 4]. Cvjetanovic et al.'s model consisted of 11 population compartments (including two for deaths from cholera or other causes, which were redundant). It did not consist of a variable for the bacterial concentration in water source. The route of cholera transmission modeled therein was "human-to-human". Cvjetanovic et al. studied the option of using vaccination, sanitation and chemoprophylaxis alone or in different combination (two or three interventions) (assumptions see Table S1).

Table S1 Assumptions made for interventions modeled in the model by Cvjetanovic et al. [3, 4].

| Interventions | Assumptions |
|---|---|
| **Vaccine** | Vaccination was carried out 21 days after the seasonal rise in the force of infection and that 75% of the population was immunized |
| **Sanitation** | A 10-year sanitation program to construct privies would gradually reduce the force of infection by 50% in 10 years among the population provided with privies. |
| **Chemoprophylaxis** | 10 close contacts per index case were treated, of whom 5 were carriers. |

# THE BASIC MODEL

Following the example of Grad et al. [5], I adopt Codeço's model [2] as the basis of our discussion, with some minor modifications:

$dS/dt = -\lambda S + \mu_b N - \mu_d S$

$dI/dt = \lambda S - \gamma I - (\mu_c + \mu_d) I$

$dR/dt = \gamma I - \mu_d R$

$dB/dt = \xi I - \delta B$

where $\lambda = \beta[B/(B+\varkappa)]$ and $N = S + I + R$

Here, S, I and R refer to susceptible, infectious and recovered populations respectively. N is the total population. B refers to the concentration of *V. cholerae* in the water reservoir or supply. The parameters are explained as in Table S2:

TABLE S2 PARAMETERS OF THE BASIC MODEL

| Parameter | Meaning |
|---|---|
| $\lambda$ | Force of infection |
| $\beta$ | "Contact rate" between the susceptible population with contaminated water |
| $\varkappa$ | Concentration of *V. cholerae* in the water reservoir that will make 50% of the susceptible population ill. |
| $\gamma$ | Recovery rate of infected people ($1/\gamma$ = duration of infectiousness) |
| $\xi$ | Rate at which infectious people contribute *V. cholerae* to the water reservoir. |

| | |
|---|---|
| **δ** | Rate at which *V. cholerae* are removed from the water reservoir. |
| $\mu_b$ | Birth rate |
| $\mu_c$ | Death rate due to cholera |
| $\mu_d$ | Death rate unrelated to cholera |

## THE FORCE OF INFECTION

The force of infection in our basic model is the per capita rate of infection experienced by susceptible individuals. It is defined as $\lambda = \beta[B/(B+\varkappa)]$, with three parameters or variables:

1. β: the "contact rate" between the susceptible population with contaminated water,
2. B: the level of contamination of the water supply (*V. cholerae* concentration), and
3. ϰ: the concentration of *V. cholerae* at which the infection rate is 50% of the maximum infection rate, that is β.

When the concentration of bacteria in the water reservoir reaches the value of ϰ, the force of infection equals half the "contact" rate of susceptible people with contaminated water (When $B = \varkappa$, $\lambda = \frac{1}{2}\beta$). When the bacterial concentration becomes very high, the force of infection equals the "contact" rate of susceptible people with contaminated water (When $B \rightarrow \infty$, $\lambda = \beta$) (See Figure S1). Its underlying assumption is a saturation effect of the bacteria concentration in the water reservoir. This is known as Monod equation in microbiology [6] and is similar to the Michaelis-Menten enzyme kinetics of single substrate [7]. It implies that if the concentration of *V. cholerae* in the water reservoir is low, its reduction by half will lead to a larger reduction in the force of infection than if it is high.

As it will be discussed in a later section of the paper, the formula for the force of infection will change if we include the so-called "human-to-human" transmission in the model.

FIGURE S1 THE ASSUMED RELATIONSHIP BETWEEN BACTERIAL CONCENTRATION IN WATER (B) AND THE FORCE OF INFECTION (λ). IF $B \rightarrow \infty$, $\lambda = \beta$. If $B = \varkappa$, $\lambda = \frac{1}{2}\beta$.

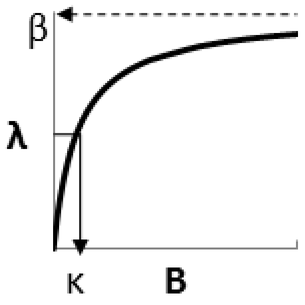


## SUMMARY OF SELECTED MODELS

Table S3 provides a summary of selected studies on their model structure, interventions studied, their predecessor models from which their models were derived and the main points of their papers.

TABLE S3 SUMMARY OF SELECTED CHOLERA TRANSMISSION DYNAMICS ORDINARY DIFFERENTIAL EQUATION MODELS

| Paper | Location | Epidemic / Endemic | Model structure | | | | Interventions studied | | | Predecessor model ^ | Main point(s) of the paper; comments. |
|---|---|---|---|---|---|---|---|---|---|---|---|
| | | | Human-to-human transmission | Hyper-infectious cholera # | Asymptomatic infected compartment | Geo-spatial simulation | Treatment | OCV | WASH | | |
| Andrews & Basu [8] | Haiti | Epidemic | No | Yes | Yes | No | Antibiotics | Yes | Clean water | Hartley et al. [9]; Miller Neilan et al. [10] | Compare effect of interventions. Model Port-au-Prince and 10 departments separately. |
| Bertuzzo et al. 2011 [11] | Haiti | Epidemic | No | No | No | Yes | No | Yes | “Sanitation” § | Bertuzzo et al. 2008 | Compare effect of interventions. Spatial model of Haiti’s >500 local communities. |
| Codeço et al.[2] | n/a | Endemic and Epidemic | No | No | No | No | No | No | No | Capasso and Paveri-Fontana [1] | Demonstrate the role of aquatic reservoir; model forced seasonal oscillation of (a) contact rate or (b) per capita contamination rate. The basis for all subsequent models |
| Date et al. [12] and Abrams et al. [13] | Haiti | Epidemic | No | No | No | Yes | No | Yes | No | Hartley et al.[9]? | The impact predicted by this model is less than the other models. The model was buried in Date et al.’s Technical Appendix 2. |
| Hartley et al. [9] | n/a | Epidemic | No | Yes | No | No | No | No | No | Codeço et al.[2] | Demonstrate the importance of hyperinfectivity of *V. cholerae* and therefore, human-to-human transmission in cholera transmission dynamics |
| King et al. [14] | Bengal* | Epidemic | Yes | No | Yes | No | No | No | No | Codeço et al.[2] | Stochastic model. Compare 4 models: 1. Basic model with waning immunity; 2. ‘Inapparent’ infection (infected but not infectious and become immune via infection); 3. Seasonality in the environmental reservoir; 4. Environmental-phage hypothesis. ‘Inapparent’ infection is important |
| Miller Neilan et al. [10] | Bangla-desh and India** | Endemic | No | Yes | Yes | No | ORT & Antibiotics | Yes | “Sanitation” § | King et al. [14] | Different optimal (most cost-effective) control measures for different locations |
| Mukandavire et al. [15] | Zimbabwe | Outbreaks in endemic area | Yes | No | No | Model each province separately | No | Yes | No | Hartley et al. [9] (similar to Tien and Earn [16]) | Estimated $R_0$ for cholera across different provinces |
| Mukandavire et al. [17] | Haiti | Epidemic | Yes | No | No | Model each 'department' separately | No | Yes | No | Mukandavire et al. [15] | Estimated $R_0$ for cholera across different 'departments' |
| Mwasa & Tchuenche [18] | n/a | Epidemic | No | No | No | No | ORT | Yes | Education, Quarantine | | Compare different interventions. Include different compartments for vaccinated, quarantined and treated individuals, as well as those who received health education regarding cholera prevention. |
| Rinaldo et al. [19] | Haiti | Epidemic | No | No | No | Yes | No | No | No | Bertuzzo et al. 2011 [11] | Rainfall-driven seasonal variation; further development of Bertuzzo et al. [11] |
| Tien & Earn† [16] | n/a | Endemic | Yes | No | No | No | No | No | No | Codeço et al.[2] | Prove that the endemic equilibrium for the model with the ‘bacteria in water’ compartment is mathematically globally stable. |
| Tien et al.[20] | London, UK‡ | Endemic | Yes | No | No | No | n/a | n/a | n/a | Tien and Earn [16] | Herald wave as a result of a new strain; Seasonality |
| Tuite et al.[21] | Haiti | Epidemic | No | No | No | Yes | No | Yes | Clean water | Tien and Earn [16] | Spatial model of Haiti’s 10 departments; compare OCV with clean water |

# It means that there is a separate compartment (and equation) for hyperinfective bacteria. ^Predecessor model is the model based on which the current model under discussion is developed. § “Sanitation” in [11] actually meant decreasing the probability of ingesting contaminated water or food (i.e. reducing $\beta$) via provision of “targeted clean water supply (through water purification systems or filters)” and health education on “hygiene and handling of water and food”. This also applied to [10]. *Historic data: British East Indian province of Bengal, 1891 – 1940. **Bogra, Bangladesh and Calcutta, India. †Tien & Earn (2010) was a general model for waterborne pathogen that applies to cholera. ‡19th century historic data. n/a: not applicable. OCV: Oral cholera vaccine. ORT: Oral rehydration treatment

# MODEL MISSPECIFICATION

In the main text, we have mentioned the challenge of model mis-specification (what Grad et al. referred as "model mis-specification" in their paper [5]), using the example of bacterial concentration vis-à-vis infectious dose. It has been pointed out (by an anonymous reviewer) that there could be a way of conversation between the two: As long as we know the size of the water reservoir and the amount of water consumed per day from that reservoir, we can estimate the infection rate (i.e. the rate of acquiring an infectious dose). While we agree that it is plausible in theory, it is very difficult in practice to estimate the size of the reservoir. More often than not, the reservoir is not well-defined. People may obtain their drinking water from multiple sources. Routine epidemiological surveys usually do not generate such data.

# PARAMETER UNCERTAINTY

To complement my discussion on parameter uncertainty in the main text, we discuss some of the sources for the values of two parameters: duration of cholera infection and cholera life span in water reservoir. We also summarize the values of the key parameters in selected models in Table S5.

## DURATION OF CHOLERA INFECTION

A number of modeling papers, for example, ref. [2, 8, 9, 13], fixed the duration of cholera infection to 5 days. We selected two and discussed their data sources here.

Hartley et al. [9] cited two references:

a) Tudor & Strato [22] (p. 313), actually referred to the International Health Regulations (1969, amended 1973) Article 62, "For the purposes of these Regulations the incubation period of cholera is five days".
b) Hendrix's review on cholera [23] (p. 1169): "In a study of 12 consecutive cholera patients in Dacca, Pakistan, in 1964, Lindenbaum and his associates found that the average duration of diarrhea was 4.7 days (range 2.7 to 6.3) and that the stool volume passed during hospitalization averaged 30.8 l. (range 5.2 to 69.1)." The data cited in Hendrix came from Table V, on page 1082 of Lindenbaum et al. [24].

The incubation period and the disease period are actually two different concepts. However, in most cholera models, incubation period is not explicitly modeled. Individuals are assumed to become infectious immediately once they are infected.

Andrews and Basu [8] cited two references:

a) Rahaman et al. [25] was a double-blinded clinical trial of doxycycline and tetracycline on cholera patients. In this trial, all participants, including those receiving the placebo, recovered within 3 to 4 days.
b) Levine et al. [26] was a cholera vaccine trial. Volunteers were observed for 96 hours (challenge studies) or 120 hours (vaccination studies) before receiving a course of tetracycline for 5 days.

These two references did not necessary support a duration of cholera infection of 5 days as used in Andrews and Basu [8].

Nonetheless, the value of 5 days for the duration of cholera infection is consistent with the data from Lindembaum et al. [24].

## CHOLERA LIFE SPAN IN WATER RESERVOIR

Complementary to the analysis of Grad et al. [5], we review here the sources for the values for cholera life span in water reservoir used in some recent models:

(a) Abrams et al. [13], Andrews and Basu [8], and Chao et al. [27] all parameterized their model with a 30-day cholera life span in water reservoir. They all cited Hartley et al. [9] as their source. In turn, Hartley et al. cited Kaper et al. [28] and the International Health Regulations (1969, amended 1973), on p. 313 in Tudor and Strati [22], but we could not identify the source in the latter reference.
(b) Bertuzzo et al. [11] cited one of their earlier papers [29] for their value of 4.39 (=1/0.228) days. It was a calibrated value in ref. [29].
(c) Tuite et al. [21] wrote that they derived their plausible range of 2.743 to 5.949 weeks – that is, 19.20 to 41.64 days – from "studies of bacterial survival in sediments", citing Hood et al. [30], which was a paper about isolation of vibrios from oysters. We are not very sure if ref. [30] was the correct reference for the purpose of Tuite et al.. The mean life span of vibrios was a parameter fitted to the Haitian cholera hospitalization data in Tuite et al. Their best fit value was 5.910 weeks (41.37 days).

Actually, data of cholera life span in water reservoir had been published as far as in the 1960s. Thanks to the suggestions of an anonymous reviewer, I hereby review some of these data for the convenience of future modeling endeavors. (Two of the sources were cited and mentioned in the discussion of Grad et al. [5].)

In 7 samples of shallow well water of pH 7.6-8.8, Felsenfeld [31] observed that Classic vibrios (13 non-haemolytic Ogawa and 5 non-haemolytic Inaba strains) survived for 7.5 ± 1.9 days, while El Tor Ogawa vibrios survived for 19.3 ± 5.1 days.

In an experiment in which water samples were inoculated with El Tor vibrios at a concentration of 1 x $10^6$ vibrios per ml, Pesigan et al. [32] (same data as in Pesigan [33]) found that vibrios survived in deep well-water sample from the Philippines for 13 days at room temperature (30˚C - 32 ˚C) and for 4 days if exposed to sunlight (daily measurement). The same vibrios were found surviving for 10 to 13 days in sea water sample at 30˚C - 32 ˚C and for 10 to 11 days if exposed to sunlight (daily measurement). In chlorinated tap water, vibrios survived for only one hour whether exposed to sunlight or not (hourly measurement) [32].

Similarly, Pandit et al. [34] measured El Tor vibrio survival in water samples from India: (a) well water from Jatauli village, Gurgagon district, Punjab, (b) well water from Bhopura village, Meerut district, Uttar Pradesh, and (c) tap water from the municipal water supply of Delhi (kept for 48 hours to remove chlorine). The water samples were inoculated with vibrios at a concentration of 1000 vibrios per ml. (mean, 992, standard derivation 15.2, range, 971-1020). It was found that a temperature of 21ºC, by day 8, vibrio concentration drops to 100 to 150 vibrios per ml. Vibrios survived for a period of (a) 18 days to (b) 51 days in well water, and (c) 12 hours in chlorine-removed tap water (measurements were made 4 times at intervals of 7 to 10 days). In the well water sample from (b) Bhopura village, vibrio concentration drops from 40 (day 19) to 20 (day 26) to 10 (day 39) per ml [34].

In another experiment, Pandit et al. [34] measured vibrio survival at an hourly basis, and observed the initial growth and subsequent decay in Bhopura well water as in Table S4.

TABLE S4 VIABILITY OF EL TOR VIBRIOS (STRAIN 205) IN BHOPURA WELL WATER (ADAPTED FROM THE FIGURE IN PANDIT ET AL. [34])

| **Hour** | **0** | **24** | **48** | **72** | **96** | **120** | **144** | **168** |
|---|---|---|---|---|---|---|---|---|
| **Number of vibrios per ml of water*** | 1000 | 4000 | ~2000 | ~750 | ~500 | ~300 | ~200 | ~200 |

* I estimated the numbers from the figure in the paper, except for 0 hour and 24 hours that were clearly stated in the text of ref. [34].

Concurring with Grad et al. [5], the uncertainty associated with the cholera life span, and therefore the bacterial decay rate, in the water reservoir, is a source of uncertainty for cholera modeling outputs.

## COMPARISON OF VALUES FOR KEY PARAMETERS ACROSS SELECTED MODELS

Furthermore, we expanded Table 1 and selected a few models that focus on the Haitian epidemic, and compared some of the parameters they used with the Codeço [2] (all converted into our notations) in Table S5. Please note that Codeço chose her parameters to illustrate her three *hypothetical* communities. She did perform sensitivity analyses on her parameters (See Table 3 of her paper).

TABLE S5 COMPARISON OF VALUES FOR KEY PARAMETERS ACROSS SELECTED MODELS

| Sym-bol | Parameters | Range found in literature, taken from ref. [5] | Codeço [2] | Abrams et al., Dec 15, 2010 [13](also [12]) | Abrams et al., Jan 28, 2011 [13] | Abrams et al., Mar 4, 2011 [13] | Andrews and Basu [8] | Bertuzzo et al. [11] | Chao et al. [27] | Tuite et al. [21] |
|---|---|---|---|---|---|---|---|---|---|---|
| β | Rate of "contact" with reservoir water ($days^{-1}$) | $10^{-5}$ to 1 | 1 (chosen for illustrating epidemic and endemic cases); 0.5 for cholera-free population. | 0.07 (Fitted to data) | 0.0301 (Fitted to data) | 0.0255 (Fitted to data) | Fitted to data | 1.0. Same as [2] | 1. Fixed. | Human-to-human transmission, Plausible range: 0.01 to 0.1 (best fit, 0.1); waterborne transmission, Plausible range: 0.789 to 0.945 (best fit, 0.944) |
| 1/γ | Duration of cholera infection (days) | 2.9 to 14 | 1/0.2 = 5 | 5 (cited [9] [29] ) | 5 | 5 | 5 (citing [25, 26]) | 1/0.2 = 5. Same as [2] | Latency period = 1 to 5 days; Infectious period = 7 to 14 days | Plausible range: 2.376 to 3.013 (best fit, 2.913) |
| 1/δ | Cholera life span in water reservoir (days, except otherwise stated) | 3 to 41 | 1/0.33 ≈ 3 | 30 [12]; 1/0.7 [13] | 1/0.033 = 30 (citing [9, 16]) | 1/0.033 = 30 | 30, citing [9] | 1/0.228, citing [29] | 30, citing [9] | Plausible range: 2.743 to 5.949 weeks (best fit, 5.910) citing [30] |
| ξ | Rate of water contamination by humans, i.e. rate of increase in *V. cholerae* concentration in the water reservoir (cells * $mL^{-1}$ * $person^{-1}$ * $day^{-1}$) | 0.01 to 10 | 10 | | | | Symptomatic patients: 1.3 * $10^{11}$ cells $person^{-1}$ $day^{-1}$; Asymptomatic patients: 1.3 * $10^{8}$ cells $person^{-1}$ $day^{-1}$ ‡ | † | 1 | n/a (they have rescaled their equation and therefore eliminate this variable, Cf. [16]) |
| κ | Concentration of cholera that yields 50% chance of infection (cells / mL) | $10^5$ to $10^6$ | $10^6$ | Population in department i / ln(population density in department i at the start of the outbreak) | Same as Dec 15 | Same as Dec 15 | Normal bacteria: $10^5$ Hyper-infectious bacteria: $10^5$ /50 = 2000 | † | 70 (source unknown) | This parameter does not exist in this model. |

Notes. †In Bertuzzo et al. (2011) [11], they normalized their equation with respect to ϰ and define a new parameter θ = p/cϰ, given c = W/N (a constant, using our notation, W = total volume of water; N = total number of people). As our parameter ξ = p/W = p/cN, therefore, p/c = ξN, so θ = ξN/ϰ. And given that in their model, $R_0 = \beta * \theta / [\delta * (\gamma + \mu_c + \mu_d)]$, using our own notations, and given the values: β = 1 $day^{-1}$; δ = 0.22 $day^{-1}$; γ = 0.20 $day^{-1}$; $\mu_c$ = 0.004 $day^{-1}$; $\mu_d$ = 0.000046 $day^{-1}$, I calculate that θ = 0.088882 $day^{-1}$. ‡ The rates of bacterial shedding had to be divided by the size of water reservoir, W = 15L * population * 365.

# VACCINATION

As discussed in the main text, one of the ways to model vaccination is to model the successfully vaccinated individuals in a separate compartment as in Figures 3 and 4. Mathematically, an equation can be added to the system (adapted from Andrews and Basu [8]):

$$dV/dt = \tau v(t) - \varepsilon V(t) - \mu V(t)$$

where,

$v(t)$: rate of individuals vaccinated per unit time;
$\tau$: percentage of vaccinated individuals that are successfully immunized (measure of direct effect of vaccines);
$1/\varepsilon$: average duration for which the successfully vaccinated individuals remain immune (e.g. 3 years); and
$\mu$: death rate.

Here the successful immunization rate is calculated by multiplying the rate of individuals vaccinated per unit time ($v(t)$) with the percentage of vaccinated individuals that are successfully immunized ($\tau$). The rate of individuals vaccinated can be varied across time as intervention program expands. Immunity will wane and successfully immunized people will become susceptible again at a rate of $\varepsilon$. As an anonymous reviewer pointed out, the direct protection of a vaccine is jointly determined by $\tau$ and $\varepsilon$. For example, Shanchol confers 66% direct protection for at least 3 years. This can happen if only 66% of the vaccinated are successfully immunized and the immunity does not wane for 3 years. Alternatively, everyone that was vaccinated might be successfully immunized but the immunity wanes so that over a period of 3 years, the cholera incidence among the vaccinated is 66% lower than that of the unvaccinated. The reality may be somewhere in between.

There are published models that studied the potential impact of oral cholera vaccines (OCV) on the Haitian epidemic. Here below we discuss the vaccination strategies modeled by some of these models and their choices of values of vaccine effectiveness.

## VACCINATION STRATEGY

Firstly, the vaccination strategies modeled by five papers on the Haitian epidemic, published in the first half of 2011, and their implication for implementation are compared in Table S6. Some of the scenarios discussed would benefit from greater consideration of implementation and logistics to develop more realistic strategies that could actually be implemented in Haiti.

TABLE S6 VACCINATION STRATEGIES MODELED FOR THE HAITIAN EPIDEMIC IN SELECTED PAPERS

| | Vaccination strategies modeled | Implication for implementation |
|---|---|---|
| Andrews and Basu [8] | 10% of the Haitian population received vaccination (about 2 million doses = 1 million individuals vaccinated) over 1 month, beginning on March 1, 2011. | Implied vaccinating around 33,000 individuals per day |
| Bertuzzo et al. [11] | (a) 300,000 doses (=150,000 people vaccinated) in Port-au-Prince within one week, starting on January 1, 2011;<br>(b) 600,000 doses (=300,000 people vaccinated), distributed uniformly across Haiti within one month, starting on November 1, 2010. | Strategy (a) and (b) implied over 20,000 and 10,000 individuals to be vaccinated per day. |
| Chao et al. [27] | (a) the hypothetical pre-vaccination, before the outbreak, in Haiti;<br>For reactive vaccinations, vaccination began 21 days after the beginning of the outbreak. 50,000 individuals were vaccinated per day thereafter:<br>(b) reactive mass vaccination;<br>(c) reactive ring vaccination; and<br>(d) reactive high-exposure vaccination (all regions along any major river in Haiti). | According to their results, among the three reactive strategies, strategy (d) is the most efficient. However, this strategy would be challenging to implement.<br>To vaccinate 50,000 individuals per day, it is the most optimistic scenario among all 4 papers discussed in this table. |
| Date et al. [12] | Use of 2-dose Dukarol for all eligible people (no prioritization)<br>Vaccination rate of 10,000 doses per day.<br>80% of those who received the first dose, receive the second dose 2 weeks after the first dose. | Vaccination rate of 10,000 doses per day (as an input to the model). |
| Tuite et al. [21] | Distributing 1 million doses (vaccinating 500,000 individuals):<br>(a) equal allocation to Haiti's 10 departments;<br>(b) allocation in proportion to the population size;<br>(c) optimized allocation (maximum reduction in total number of cases).<br>Comparing the results, if vaccination was completed from days 1, 30, 60, 90, 120, and 150 from 21 October 2010. | The key result was that the earlier the vaccination campaign was completed, the more cases were averted. Optimization would begin to make a difference when vaccination started late.<br>The vaccination schedule under consideration imply vaccinating per day:<br>Completion on Day 30: 16667<br>Completion on Day 60: 8333<br>Completion on Day 90: 5556<br>Completion on Day 120: 4167<br>Completion on Day 150: 3333 |

## VACCINE EFFECTIVENESS

Oral cholera vaccine effectiveness empirical data are listed in Table S7.

TABLE S7 EMPIRICAL DATA OF ORAL CHOLERA VACCINE EFFECTIVENESS AGAINST SYMPTOMATIC CHOLERA WITH CONFIRMED INFECTION.

| Oral Cholera Vaccine | Vaccine effectiveness against symptomatic cholera with confirmed infection, $VE_{SP}$ (Direct effect) | Location of the vaccine trial | References |
|---|---|---|---|
| **Dukarol** | 55% in 1 year | Matlab, Bangladesh | Ali et al. [35] |
| **Dukarol** | 79% in 15 months | Zanzibar, Tanzania | Khatib et al. [36] |
| **Shanchol** | 66% in 3 years | Kolkata, India | Sur et al. [37] |
| **Shanchol** | 65% in 5 years | Kolkata, India | Bhattacharya et al. [38] |

Using the symbols as in Chao et al.[27], we summarize the vaccine effectiveness measures used in the five published models on the Haitian epidemic in Table S8.

TABLE S8 VACCINE EFFECTIVENESS MEASURES IN FIVE SELECTED MODELS ON THE HAITIAN EPIDEMIC

| | **Vaccine Effectiveness to prevent infection from exposure ($VE_S$)** | **Vaccine Effectiveness to prevent infected individuals from being symptomatic ($VE_P$)** | **Vaccine Effectiveness in reducing infectiousness of infectious individuals ($VE_I$)** | **Mean duration of immunity** | **Notes** |
|---|---|---|---|---|---|
| **Andrews and Basu [8]** | 67% [39, 40] (2-dose) | 0% | 0% | 2 years [39, 40]; (sensitivity analysis: 0.5 to 5 years) | "All or none": either 100% immune or susceptible |
| **Bertuzzo et al. [11]** | Assumed to be 100% * (2-dose) | 0% | 0% | Longer than the model's timeframe (6 months) | Judgment based on their model structure. |
| **Chao et al. [27]** | Assumed to be 0, citing [41] † | 64%, citing [41] (2-dose) | 50%, citing [42] (2-dose) | Longer than the model's timeframe (6 months) | Vaccine effectiveness rises from 0 to 50% of its full value for the first 7 days (mimic first dose), and then from 50% to 100% from day 7 to day 21 (mimic second dose). ‡ |
| **Date et al. [12]** | 50% (1-dose) at 2 wk; 85% (2-dose) from 8.5d to 6mo; ~62% at 1yr; 58% at 2yr; 18% at 3yr | 0% | 0% | - | Immunity wanes according to an exponential decay regression curve fit. |
| **Tuite et al. [21]** | 100% (2-dose) | 0% | 0% | Longer than the model's timeframe (4 months) | There is a probability of vaccination within a department. But once an individual is vaccinated (assumed two doses), he is immune. § |

*Unless one get infected before they turn immune. †So that the vaccine effectiveness against symptomatic cholera with confirmed infection, $VE_{SP}$, = 1 – (1 – $VE_S$) * (1 – $VE_P$) = 64% [41]. ‡There is no evidence that people vaccinated with one dose will have any protection. We suggest future models assume no immunity until the time when the second dose is supposed to be administered. § While Tuite et al. mentioned a "50% effective vaccine", citing the Cochrane review for injected cholera vaccines [43], they do not take the less-than-perfect vaccine effectiveness into account.

Three out of the 5 papers under discussion use a more reasonable vaccine effectiveness estimate. Chao et al. [27] is the most sophisticated one, separating the VE on susceptibility-becoming symptomatic and the VE on reducing infectiousness. We have to emphasize the point that to assume the direct VE as 100% (as in ref. [11, 21]) will greatly over-estimate the impact of vaccination.

Bertuzzo et al. [11] created an extra variable / compartment for susceptible individuals who have received two doses of OCV but have not yet developed immunity and therefore are still susceptible to infection. Therefore, they created two input parameters: vaccination rate (number of vaccine doses administered per day), which was a logistical parameter, and a rate of vaccinated individuals becoming immune. While they acknowledged that "full immunity requires 7-10 days after the second dose to build up", they used an estimation of a rate of 0.5 $day^{-1}$ for those individuals who had received two doses of vaccine to become immune. Perhaps 1/7 to 1/10 would be a better input. Given the very short period of time an individual stays in that compartment, its impact may not be significant. More importantly, based on their model structure, we can tell that they assume nearly everyone who is vaccinated will turn immune from infection eventually, except those who are vaccinated, and then infected, before they turn immune. We know that cholera vaccine effectiveness is less than 100%. This may not be a correct assumption. This may be compensated mathematically by the fact that they allow vaccinated individuals to remain susceptible for a while (a mean of 2 days).

# WATER, SANITATION AND HYGIENE

Water, sanitation and hygiene can be represented in the basic model as follows:

$dB/dt = (1\text{-}san)*\xi*I - (1+sou)*\delta*B$

$\lambda = (1\text{-}h)*\beta*(1\text{-}p)*B/[(1\text{-}p)*B+\varkappa]$

where,

san: sanitation interventions and health promotion of their utilization and hand hygiene

sou: treatment of water at source (e.g. chlorination of piped water)

p: point-of-use water purification (via boiling, chlorination or filters)

h: using alternative source of drinking water

However, one potential critique of this proposal is that we usually do not measure the rate of bacterial removal from water. For example, we may measure the compliance of point-of-use chlorination water purification by measuring the chlorine residual in the drinking water. Those data are usually given in binary form (below or above a threshold that is considered "safe" water).

# ASYMPTOMATIC INFECTIONS

Bertuzzo et al. [11] handled the issue of underreporting by assuming that only 5% of cholera cases reported to any healthcare facilities. This was estimated by assuming that 25% of infected individuals were symptomatic, among which 20% developed acute diarrhea and required medical attention. They included the underreporting scaling factor in their uncertainty analysis. (See Auxiliary materials of ref. [11]).

In order to explain the rapid reduction from an estimated $R_0$ of 2.78 or 2.90 at the beginning of the outbreak to an estimated effective reproduction number ($R_e$) of 0.5 after three months, Tuite et al. [21] concluded that it was the result of the public health response in the early phase of the epidemic. Rinaldo et al. [44] criticized Tuite et al. for not including asymptomatic infections in their model and therefore over-estimated the impact of the interventions (a 6-fold decrease in $R_e$). Instead, they proposed an alternative explanation by taking asymptomatic infections (and subsequent depletion of the susceptible population) into account. Tuite et al. countered by stating that Rinaldo et al. confused the two distinct concepts of $R_0$ and $R_e$ and that the observed surge in cholera incidence in May 2011 was a proof that the susceptible population has not yet been depleted. (Rinaldo et al. argued that their explanation "does not require reproduction numbers to decrease with time"[44]; however, Tuite et al. did confuse the two concepts themselves when they wrote "a decrease in R0 by an average of 1.8% per day" in their original paper [21].) Tuite et al. could have included a scaling factor to take into account the underreporting of cases (asymptomatic cases and those symptomatic case who were unable to reach healthcare facilities to be "reported") as they fit their modeling output to observed data. But this would probably give them an even higher $R_0$. But more importantly, the size of the reduction in effective reproduction number might not be as big as Tuite et al. originally reported[21], and this would make the estimates closer towards their estimated effect of interventions. Likewise, while there must have been a reasonable size of population who had asymptomatic infection and became immune to cholera, herd immunity through infection would be unlikely to be the sole explanation for the reduction of the effective reproduction number. Apparently, the provision of clean water and prompt treatment of symptomatic cases greatly reduced cholera incidence in some camps of displaced populations. Rinaldo et al. [44] might have overstated the impact of asymptomatic infection (the compartment of susceptible was "depleted"). Apparently, the surge in incidence in mid-2011 was recognized by a later paper of theirs [19].

In summary, underreporting of cases, including asymptomatic cases, should be taken into account when fitting modeling outputs to observed data (even if the model does not have a distinct compartment for asymptomatic cases). Nonetheless, the

reduction in effective reproduction number during the first three months of the epidemic in Haiti cannot be solely explained by the depletion of susceptible through infection.

## HYPERINFECTIOUS BACTERIA AND "HUMAN-TO-HUMAN" TRANSMISSION

Hartley et al. [9] was the first to introduce hyperinfectious bacteria into a cholera model. They introduced a variable for hyperinfectious *V. cholerae* ($B_H$) with this equation:

$dB_H/dt = \xi I - \chi B_H$

where $\chi$ is the rate that $B_H$ lose their hyperinfectivity and become normal bacteria.

The force of infection is formulated as such [9]:

$\lambda = \beta_w[B_L/(B_L+\varkappa)] + \beta_h[B_H/(B_H+\varkappa)]$

However, the hyperinfectious state is very brief (5 to 24 hours) while the dynamics of infection and recovery is in a scale of days. As Pascual et al. [45] explained, the number of infectious individuals (I) will essentially be a 'constant' to $B_H$ within their short 'lifespan'. They will be in a quasi-equilibrium where we can track $B_H$ with I. Therefore, instead of having a separate variable, $B_H$, we can model the impact of hyperinfectious bacteria as if it is "human-to-human" transmission, by adding a transmission term to the composition of the force of infection, e.g.:

$\lambda = \beta_w[B/(B+\varkappa)] + \beta_h I$

where the transmission coefficients ("contact" rate) of water-borne and "human-to-human" transmission are represented as $\beta_w$ and $\beta_h$ respectively. The "human-to-human" transmission component in the force of infection equation is proportional to the number of infected individuals (at a particular time, t). It varies with incidence.

Behind the equation is the 'random mixing' assumption, which is similar to the mass action principle in chemistry. As susceptible people are infected at a rate of $\lambda S$, the "human-to-human" transmission term $\beta_h IS$ implies that in a homogeneous population, infected and susceptible populations are randomly mixed, and the per capita rate of transmission is $\beta_h$.

## CHOICE OF PARAMETERS

Here I discussed the choice of the parameters related to hyperinfectivity in the context of the original experiments from which the parameters were drawn.

Hartley et al. [9] used a scaling factor of 700 for hyperinfectivity, i.e. the infectious dose of 50% chance of infection ($IC_{50}$) of hyperinfectious *V. cholerae* is 700 times of that for normal *V. cholerae*. They cited the original experimental work of Merrell et al. [46]. However, Merrell et al.'s paper notes, "As shown in Fig. 1a [of their paper], *V. cholerae* shed from the human gastrointestinal tract (human-shed) showed greatly enhanced infectivity, out-competing the in vitro-grown strain by **as much as** 700-fold" (bold underlined emphasis mine). In other words, the maximum is 700-fold. By visual inspection of Figure 1a of Merrell et al., the geometric means for experiment 1, 2 and 3, were ~10, 10-20, ~200, respectively. It may not be prudent to fix the scaling factor to the maximum value of 700. A sensitivity analysis on this scaling factor should be performed and its results presented, but this was not found in Hartley et al. [9].

The scaling factor of 50 in Andrews and Basu's [8] and that of 100 in Chao et al. [27] for hyperinfectivity are more in line with both ref. [46] and [47]. In Alam et al.'s [47] mouse-passage model of hyperinfectious *V. cholerae*, the $IC_{50}$ for mouse-passaged bacteria and that for bacteria grown in vitro are around 50 and 1000 colony-forming units (c.f.u.) respectively (in this case, percentage of mice infected after 24 hours, see Figure 3 of ref. [47]). And the competitive index from competition assays infant mice (as in Figures 1 and 2 of ref. [47]), hyperinfectious bacteria are either slightly higher or lower than 100-fold more infectious than non-hyperinfectious bacteria. Therefore, Andrews and Basu used a more conservative value that are more in line with experimental results than that of Hartley et al. [9].

With regard to the duration of hyperinfectious state, Merrell et al. [46] presented experimental results that after 5 hours in the environment, bacteria freshly shed by humans did exist in their hyperinfectious state but not after 18 hours. Alam et al. showed that mouse-passaged bacteria showed no difference in colonization of mice's small intestines, compared with those grown in vitro, 24 hours post-inoculation. However, they showed that 5 hours after inoculation, mouse-passaged bacteria showed more rapid replication than those grown in vitro. Therefore, one could say that Andrews and Basu [8] used the longest possible duration supported by experiment (24 hours), while Hartley et al. [9] used the more conservative value of 5 hours.

## SPATIAL ELEMENTS: HUMAN MOVEMENT & RIVER NETWORK

In all the models introduced in the above sections, the element of time is paramount, as all variables vary with time. However, there are no spatial elements in these models. In this section, I shall briefly discuss some examples of models that handle these issues.

**Human movement** is important in cholera transmission, as infected individuals shed bacteria into the environment through which they travel. One way to incorporate this element is to include in the force of infection term of region *i*, different transmission terms that represent transmission from region *j* to *i*. Such terms can be a function of the population sizes of the regions and their distance between them. An example is a paper by Tuite et al. [21] in which the 10 departments of Haiti were modeled explicitly. A similar approach was adopted by Bertuzzo et al. [11] but with a greater geographical resolution (>500 local communities, at the fourth administrative level in Haiti).

**River networks** contribute to cholera transmission. As illustrated in the first week of the cholera outbreak in Haiti in October 2010, an outbreak that began in the upper Arbonite River spread along the river downstream to the towns at the estuary.

Recently, modelers combine both elements of human movement and river networks into a single model and expanded our understanding of spatial elements in cholera epidemics.

An example is an agent-based model by Chao et al. [27] (Figure S2), where they group individuals into households, and households into communities (of roughly 500 people each). They explicitly model daily commuting for workers between communities in Haiti. Two modes of long-distance travel were included, namely highway travel and non-highway travel. They also explicitly modeled cholera transmission via network by making the rivers as a second environment reservoir. Infectious individuals in one community shed bacteria into their own community local water source as well as to the river where the bacteria were transported downstream (with some loss) to another community.

FIGURE S2 A CARTOON OF CHOLERA TRANSMISSION VIA RIVER FLOW (ADAPTING THE IDEA FROM CHAO ET AL., 2011[27]) S: SUSCEPTIBLE; I: INFECTED; R: RECOVERED; B: BACTERIA IN LOCAL WATER SOURCE; BR: BACTERIA IN RIVER

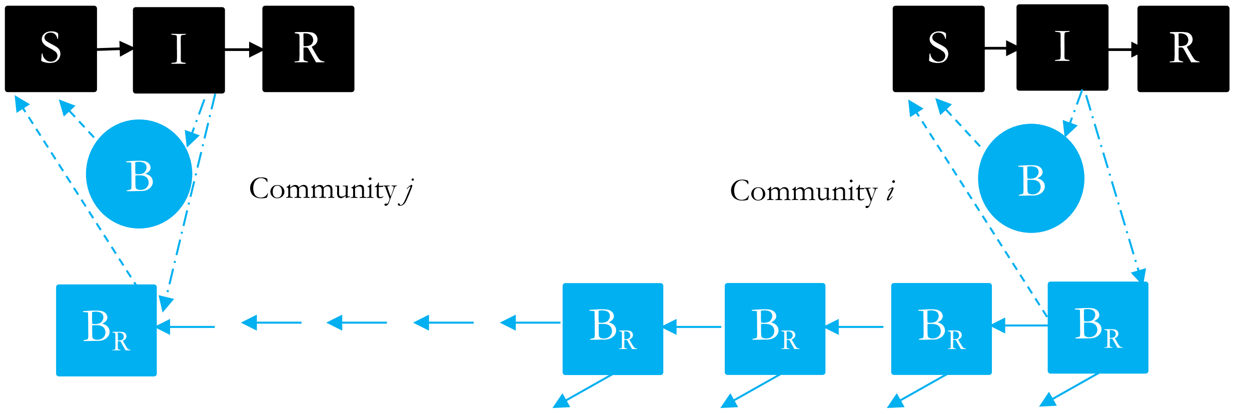


Rinaldo and his research group have produced a stream of recent articles that tackle river network. Based on Bertuzzo et al. [11], Rinaldo et al. combined human movement, river flow and seasonal rainfall data of Haiti into their model and fit their model to Haiti epidemic data and made prediction of the incidence curve up to January 2014 [19]. Mari et al. studied the interaction between human mobility with waterways and sanitation coverage in KwaZulu-Natal, South Africa [48].

## CLIMATE & SEASONALITY

Seasonality is known to play a role in cholera incidence pattern. Climatic factors, like rainfall patterns and El Nino-Southern Oscillation [49, 50], are known as drivers of cholera seasonal patterns.

Pascual et al. [51] proposed an ODE model to study how rainfall (and water volume in the rivers) affects cholera dynamics. They replaced the equation for bacterial concentration in water (as in the basic model by Codeço [2]) with 2 equations: one for water volume and the other for "fomites" or bacterial abundance. The equations were written so that when water volumes are low, the force of infection will be higher than when they are high. The bacterial concentration can be changed directly by climatic factors without affecting its growth rate [51]. Seasonal rainfall patterns have been observed to correlate with the timing of cholera outbreaks in endemic areas. This component was added by Rinaldo et al. to their model [19]. Reiner et al. used a multidimensional inhomogeneous Markov chain model to study both the climatic effect and spatial element [52].

Tien et al. used sinusoidal forcing in the "contact rate" between susceptible population and contaminated water to model seasonality in cholera transmission as observed in 19th century London [20].

Temporary immunity after infection as a factor has also been proposed. A semi-parametric method to fit a model of two difference equations (Susceptible and Infected individuals) to historic incidence data taking into account temporary immunity and seasonality have been developed by Koelle and colleagues [53, 54].

# CHOLERA MODELING APPLIED IN AN OUTBREAK SCENARIO: ABRAMS ET AL. AS AN EXAMPLE

To illustrate the considerations when one constructs a model for a specific purpose, we use the model written by Joseph Abrams and colleagues [13] as an example. Among all published models on the Haitian cholera outbreak, Abrams et al.'s model may have the highest policy relevance, as modeling outputs were conveyed directly to policy-makers and non-governmental organizations in real time during the outbreak. We approach this by looking at some of the features of the model structure, presenting the reason for their inclusion or exclusion by Abrams et al., and comments on their choice in Table S8. Figure S3 provides an illustration of the model structure.

TABLE S8 SELECTED COMPONENTS INCLUDED OR EXCLUDED IN THE CDC MODEL BY ABRAMS ET AL.[13]

| Components | Incorporated (Yes/No) | Reason given by Abrams and colleagues | Comments |
|---|---|---|---|
| Only a fraction of the Total population being Susceptible | Yes | "Not all people are expected to be uniformly exposed to the cholera epidemic; people who have access to clean water and safe sanitation, or are geographically isolated from the outbreak may be effectively protected from infection". [13] | What the model does is to stratify the population into 2 groups, one can be exposed and one cannot. Susceptible people are being moved from the latter to the former at a rate. This necessitates an additional parameter that depends on model fit. No data other than the incidence data are used to support the value of the parameter. |
| Two "Removed" compartments – R1 and R2 | Yes | Population recovered from cholera would remain "fully immune for 6 months before slowly losing immunity at the same rate as measured in a large-scale vaccine study" [13] | This is a strategy to alleviate the problem of having an exponential declining flow of people losing their immunity. (The equation represented by the arrow will imply exponential "decay") |
| Seasonal changes | No | "There is no recent experience with cholera in the Caribbean" [13] | To keep the model simple, seasonality (especially rainfall pattern) is left out. Therefore it will not be possible to model the summer 2011 and summer 2012 peaks of cholera incidence. |
| Spatial component (department-specific); e.g. geographical connected ness: infected people in a neighbouring department can contaminate water in a department; separate "water initiation parameter" for departments affected later in the epidemic | Yes | Used an early fit values, without providing reason why they include this component. | No data support their values apart from fitting to the incidence data. |
| Different "kappa" for different departments: population/ln(density) | Yes | "Early model testing showed that rates of disease spread within departments were positively associated with population density, and scaling water infectivity by log population density [ln($dens_i$)] was shown to improve model fit." | Abrams et al. have access to Haitian data, broken down for Port-au-Prince and the 10 departments. Therefore they can fit the model to the data by 11 geographical units. |

As illustrated in Figure S3, in each department (Haiti's political division), there are 5 population compartments (black boxes) and a compartment for bacterial concentration in drinking water sources (blue oval). Different from the models that were

previously introduced in this paper, Abrams et al. created a "Total population" compartment so that they could adjust the fraction of population that was at risk of infection along the time line as cholera spreads from one department to another (spatial element). They also incorporated two Removed compartments to incorporate a delay in waning immunity. Abrams et al.'s model incorporated the spatial heterogeneity of the outbreak by incorporating the bacterial shedding of infectious population into other compartments (population movement) and the transfer of bacteria from one compartment to another (hydraulic movement).

Abrams et al.'s model was a difference equation model – instead of differential equations. It was written in R, an open-source free software, that is readily available.

As Abrams et al. explained [13], there is collinearity between certain parameters. Therefore they fixed some of them at values that were fitted from early iterations of the model. However, their paper avoided answering an important question: why certain variables ("compartments") or parameters are needed at the first place. While introducing new structures to the model may increase the fit to the data, the inclusion of parameters that are not supported by empirical data (apart from fitting to the incidence data) may create unnecessary uncertainty for the model. The fact that there is much collinearity between parameters suggests the possibility of redundancy in the model.

By deploying their model, Abrams et al. provided timely estimates to policy-makers and other stakeholders. In this sense, they did a successful job despite all the caveats of their model.

FIGURE S3 THE ABRAMS ET AL.'S MODEL (ADAPTED FROM REF.[13]). NOTE: A DEPARTMENT IN HAITI REFERS TO AN ADMINISTRATIVE AREA SIMILAR TO A PROVINCE IN OTHER COUNTRIES.

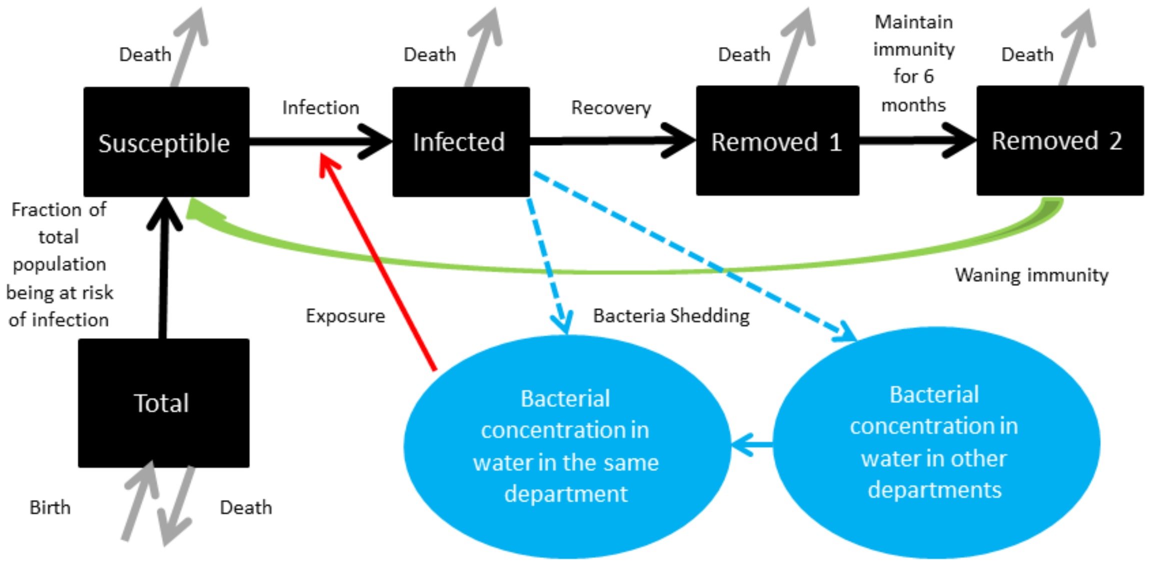

# REFERENCES


1. Capasso V, Paveri-Fontana SL: **A mathematical model for the 1973 cholera epidemic in the European Mediterranean region**. *Revue d'epidemiologie et de sante publique* 1979, **27**(2):121-132.
2. Codeco CT: **Endemic and epidemic dynamics of cholera: the role of the aquatic reservoir**. *BMC infectious diseases* 2001, **1**:1.
3. Cvjetanovic B, Grab B, Uemura K: **Dynamics of acute bacterial diseases. Epidemiological models and their application in public health. Part I. Theory and practice of epidemiological models**. *Bulletin of the World Health Organization* 1978, **56 Suppl 1**:9-23.
4. Cvjetanovic B, Grab B, Uemura K: **Dynamics of acute bacterial diseases. Epidemiological models and their application in public health. Part II. Epidemiological models of acute bacterial diseases**. *Bulletin of the World Health Organization* 1978, **56 Suppl 1**:25-143.
5. Grad YH, Miller JC, Lipsitch M: **Cholera modeling: challenges to quantitative analysis and predicting the impact of interventions**. *Epidemiology* 2012, **23**(4):523-530.
6. Monod J: **The Growth of Bacterial Cultures**. *Annual Review of Microbiology* 1949, **3**:371-394.
7. Chen WW, Niepel M, Sorger PK: **Classic and contemporary approaches to modeling biochemical reactions**. *Genes & development* 2010, **24**(17):1861-1875.
8. Andrews JR, Basu S: **Transmission dynamics and control of cholera in Haiti: an epidemic model**. *Lancet* 2011, **377**(9773):1248-1255.
9. Hartley DM, Morris JG, Jr., Smith DL: **Hyperinfectivity: a critical element in the ability of V. cholerae to cause epidemics?** *PLoS medicine* 2006, **3**(1):e7.
10. Miller Neilan RL, Schaefer E, Gaff H, Fister KR, Lenhart S: **Modeling optimal intervention strategies for cholera**. *Bulletin of mathematical biology* 2010, **72**(8):2004-2018.
11. Bertuzzo E, Mari L, Righetto L, Gatto M, Casagrandi R, Blokesch M, Rodriguez-Iturbe I, Rinaldo A: **Prediction of the spatial evolution and effects of control measures for the unfolding Haiti cholera outbreak**. *Geophys Res Lett* 2011, **38**:L06403.
12. Date KA, Vicari A, Hyde TB, Mintz E, Danovaro-Holliday MC, Henry A, Tappero JW, Roels TH, Abrams J, Burkholder BT *et al*: **Considerations for oral cholera vaccine use during outbreak after earthquake in Haiti, 2010-2011**. *Emerg Infect Dis* 2011, **17**(11):2105-2112.
13. Abrams JY, Copeland JR, Tauxe RV, Date KA, Belay ED, Mody RK, Mintz ED: **Real-time modelling used for outbreak management during a cholera epidemic, Haiti, 2010-2011**. *Epidemiology and infection* 2013, **141**(6):1276-1285.
14. King AA, Ionides EL, Pascual M, Bouma MJ: **Inapparent infections and cholera dynamics**. *Nature* 2008, **454**(7206):877-880.
15. Mukandavire Z, Liao S, Wang J, Gaff H, Smith DL, Morris JG, Jr.: **Estimating the reproductive numbers for the 2008-2009 cholera outbreaks in Zimbabwe**. *Proc Natl Acad Sci U S A* 2011, **108**(21):8767-8772.
16. Tien JH, Earn DJ: **Multiple transmission pathways and disease dynamics in a waterborne pathogen model**. *Bulletin of mathematical biology* 2010, **72**(6):1506-1533.
17. Mukandavire Z, Smith DL, Morris JG, Jr.: **Cholera in Haiti: Reproductive numbers and vaccination coverage estimates**. *Scientific reports* 2013, **3**:997.
18. Mwasa A, Tchuenche JM: **Mathematical analysis of a cholera model with public health interventions**. *Bio Systems* 2011, **105**(3):190-200.
19. Rinaldo A, Bertuzzo E, Mari L, Righetto L, Blokesch M, Gatto M, Casagrandi R, Murray M, Vesenbeckh SM, Rodriguez-Iturbe I: **Reassessment of the 2010-2011 Haiti cholera outbreak and rainfall-driven multiseason projections**. *Proc Natl Acad Sci U S A* 2012, **109**(17):6602-6607.
20. Tien JH, Poinar HN, Fisman DN, Earn DJ: **Herald waves of cholera in nineteenth century London**. *Journal of the Royal Society, Interface / the Royal Society* 2011, **8**(58):756-760.
21. Tuite AR, Tien J, Eisenberg M, Earn DJ, Ma J, Fisman DN: **Cholera epidemic in Haiti, 2010: using a transmission model to explain spatial spread of disease and identify optimal control interventions**. *Ann Intern Med* 2011, **154**(9):593-601.
22. Tudor V, Strati I: **Smallpox: Cholera**. Tunbridge Wells, Kent: Abacus Press; 1977.
23. Hendrix TR: **The pathophysiology of cholera**. *Bulletin of the New York Academy of Medicine* 1971, **47**(10):1169-1180.
24. Lindenbaum J, Greenough WB, 3rd, Benenson AS, Oseasohn R, Rizvi S, Saad A: **Non-Vibrio Cholera**. *Lancet* 1965, **285**(7395):1081-1083.

25. Rahaman MM, Majid MA, Alam A, Islam MR: **Effects of doxycycline in actively purging cholera patients: a double-blind clinical trial**. *Antimicrobial agents and chemotherapy* 1976, **10**(4):610-612.
26. Levine MM, Kaper JB, Herrington D, Losonsky G, Morris JG, Clements ML, Black RE, Tall B, Hall R: **Volunteer studies of deletion mutants of Vibrio cholerae O1 prepared by recombinant techniques**. *Infection and immunity* 1988, **56**(1):161-167.
27. Chao DL, Halloran ME, Longini IM, Jr.: **Vaccination strategies for epidemic cholera in Haiti with implications for the developing world**. *Proc Natl Acad Sci U S A* 2011, **108**(17):7081-7085.
28. Kaper JB, Morris JG, Jr., Levine MM: **Cholera**. *Clinical microbiology reviews* 1995, **8**(1):48-86.
29. Bertuzzo E, Azaele S, Maritan A, Gatto M, Rodriguez-Iturbe I, Rinaldo A: **On the space-time evolution of a cholera epidemic**. *Water Resour Res* 2008, **44**(1):W01424.
30. Hood MA, Ness GE, Rodrick GE: **Isolation of Vibrio cholerae serotype O1 from the eastern oyster, Crassostrea virginica**. *Applied and environmental microbiology* 1981, **41**(2):559-560.
31. Felsenfeld O: **Some observations on the cholera (El Tor) epidemic in 1961-62**. *Bull World Health Organ* 1963, **28**(3):289-296.
32. Pesigan TP, Plantilla J, Rolda M: **Applied studies on the viability of El Tor vibrios**. *Bull World Health Organ* 1967, **37**(5):779-786.
33. Pesigan TP: **Studies on the Viability of El Tor Vibrios in Contaminated Foodstuffs, Fomites, and Water**. In: *Proceedings of the Cholera Research Symposium.* edn. Edited by Bushnell OA, Brookhyser CS. Hawaii: U.S. Public Health Service; 1965.
34. Pandit CG, Pal SC, Murti GV, Misra BS, Murty DK, Shrivastav JB: **Survival of Vibrio cholerae biotype El Tor in well water**. *Bull World Health Organ* 1967, **37**(4):681-685.
35. Ali M, Emch M, von Seidlein L, Yunus M, Sack DA, Rao M, Holmgren J, Clemens JD: **Herd immunity conferred by killed oral cholera vaccines in Bangladesh: a reanalysis**. *Lancet* 2005, **366**(9479):44-49.
36. Khatib AM, Ali M, von Seidlein L, Kim DR, Hashim R, Reyburn R, Ley B, Thriemer K, Enwere G, Hutubessy R *et al*: **Effectiveness of an oral cholera vaccine in Zanzibar: findings from a mass vaccination campaign and observational cohort study**. *Lancet Infect Dis* 2012, **12**(11):837-844.
37. Sur D, Kanungo S, Sah B, Manna B, Ali M, Paisley AM, Niyogi SK, Park JK, Sarkar B, Puri MK *et al*: **Efficacy of a low-cost, inactivated whole-cell oral cholera vaccine: results from 3 years of follow-up of a randomized, controlled trial**. *PLoS Negl Trop Dis* 2011, **5**(10):e1289.
38. Bhattacharya SK, Sur D, Ali M, Kanungo S, You YA, Manna B, Sah B, Niyogi SK, Park JK, Sarkar B *et al*: **5 year efficacy of a bivalent killed whole-cell oral cholera vaccine in Kolkata, India: a cluster-randomised, double-blind, placebo-controlled trial**. *The Lancet infectious diseases* 2013, **13**(12):1050-1056.
39. Sur D, Lopez AL, Kanungo S, Paisley A, Manna B, Ali M, Niyogi SK, Park JK, Sarkar B, Puri MK *et al*: **Efficacy and safety of a modified killed-whole-cell oral cholera vaccine in India: an interim analysis of a cluster-randomised, double-blind, placebo-controlled trial**. *Lancet* 2009, **374**(9702):1694-1702.
40. Thiem VD, Deen JL, von Seidlein L, Canh do G, Anh DD, Park JK, Ali M, Danovaro-Holliday MC, Son ND, Hoa NT *et al*: **Long-term effectiveness against cholera of oral killed whole-cell vaccine produced in Vietnam**. *Vaccine* 2006, **24**(20):4297-4303.
41. Black RE, Levine MM, Clements ML, Young CR, Svennerholm AM, Holmgren J: **Protective efficacy in humans of killed whole-vibrio oral cholera vaccine with and without the B subunit of cholera toxin**. *Infection and immunity* 1987, **55**(5):1116-1120.
42. Longini IM, Jr., Nizam A, Ali M, Yunus M, Shenvi N, Clemens JD: **Controlling endemic cholera with oral vaccines**. *PLoS medicine* 2007, **4**(11):e336.
43. Graves PM, Deeks JJ, Demicheli V, Jefferson T: **Vaccines for preventing cholera: killed whole cell or other subunit vaccines (injected)**. *Cochrane Database Syst Rev* 2010(8):CD000974.
44. Rinaldo A, Blokesch M, Bertuzzo E, Mari L, Righetto L, Murray M, Gatto M, Casagrandi R, Rodriguez-Iturbe I: **A transmission model of the 2010 cholera epidemic in Haiti**. *Ann Intern Med* 2011, **155**(6):403-404; author reply 404.
45. Pascual M, Koelle K, Dobson AP: **Hyperinfectivity in cholera: a new mechanism for an old epidemiological model?** *PLoS medicine* 2006, **3**(6):e280.
46. Merrell DS, Butler SM, Qadri F, Dolganov NA, Alam A, Cohen MB, Calderwood SB, Schoolnik GK, Camilli A: **Host-induced epidemic spread of the cholera bacterium**. *Nature* 2002, **417**(6889):642-645.
47. Alam A, Larocque RC, Harris JB, Vanderspurt C, Ryan ET, Qadri F, Calderwood SB: **Hyperinfectivity of human-passaged Vibrio cholerae can be modeled by growth in the infant mouse**. *Infection and immunity* 2005, **73**(10):6674-6679.

48. Mari L, Bertuzzo E, Righetto L, Casagrandi R, Gatto M, Rodriguez-Iturbe I, Rinaldo A: **Modelling cholera epidemics: the role of waterways, human mobility and sanitation**. *Journal of the Royal Society, Interface / the Royal Society* 2012, **9**(67):376-388.
49. Pascual M, Rodo X, Ellner SP, Colwell R, Bouma MJ: **Cholera dynamics and El Nino-Southern Oscillation**. *Science* 2000, **289**(5485):1766-1769.
50. Rodo X, Pascual M, Fuchs G, Faruque AS: **ENSO and cholera: a nonstationary link related to climate change?** *Proc Natl Acad Sci U S A* 2002, **99**(20):12901-12906.
51. Pascual M, Bouma MJ, Dobson AP: **Cholera and climate: revisiting the quantitative evidence**. *Microbes and infection / Institut Pasteur* 2002, **4**(2):237-245.
52. Reiner RC, Jr., King AA, Emch M, Yunus M, Faruque AS, Pascual M: **Highly localized sensitivity to climate forcing drives endemic cholera in a megacity**. *Proc Natl Acad Sci U S A* 2012, **109**(6):2033-2036.
53. Koelle K, Pascual M: **Disentangling extrinsic from intrinsic factors in disease dynamics: a nonlinear time series approach with an application to cholera**. *The American naturalist* 2004, **163**(6):901-913.
54. Koelle K, Rodo X, Pascual M, Yunus M, Mostafa G: **Refractory periods and climate forcing in cholera dynamics**. *Nature* 2005, **436**(7051):696-700.
